# Metformin in Antiviral Therapy: Evidence and Perspectives

**DOI:** 10.3390/v16121938

**Published:** 2024-12-18

**Authors:** Iryna Halabitska, Pavlo Petakh, Oleh Lushchak, Iryna Kamyshna, Valentyn Oksenych, Oleksandr Kamyshnyi

**Affiliations:** 1 Department of Therapy and Family Medicine, I. Horbachevsky Ternopil National Medical University, Voli Square, 1, 46001 Ternopil, Ukraine; 2 Department of Biochemistry and Pharmacology, Uzhhorod National University, 88017 Uzhhorod, Ukraine; 3 MRC Laboratory of Medical Sciences, London W12 0HS, UK; 4 Department of Medical Rehabilitation, I. Horbachevsky Ternopil National Medical University, 46001 Ternopil, Ukraine; kamyshna_ii@tdmu.edu.ua; 5 Department of Clinical Science, University of Bergen, 5020 Bergen, Norway; 6 Department of Microbiology, Virology, and Immunology, I. Horbachevsky Ternopil National Medical University, 46001 Ternopil, Ukraine

**Keywords:** metformin, broad-spectrum antiviral, AMPK activation, mTOR inhibition, viral replication, oxidative stress, inflammation

## Abstract

Metformin, a widely used antidiabetic medication, has emerged as a promising broad-spectrum antiviral agent due to its ability to modulate cellular pathways essential for viral replication. By activating AMPK, metformin depletes cellular energy reserves that viruses rely on, effectively limiting the replication of pathogens such as influenza, HIV, SARS-CoV-2, HBV, and HCV. Its role in inhibiting the mTOR pathway, crucial for viral protein synthesis and reactivation, is particularly significant in managing infections caused by HIV, CMV, and EBV. Furthermore, metformin reduces oxidative stress and reactive oxygen species (ROS), which are critical for replicating arboviruses such as Zika and dengue. The drug also regulates immune responses, cellular differentiation, and inflammation, disrupting the life cycle of HPV and potentially other viruses. These diverse mechanisms suppress viral replication, enhance immune system functionality, and contribute to better clinical outcomes. This multifaceted approach highlights metformin’s potential as an adjunctive therapy in treating a wide range of viral infections.

## 1. Introduction

**Figure 1:**
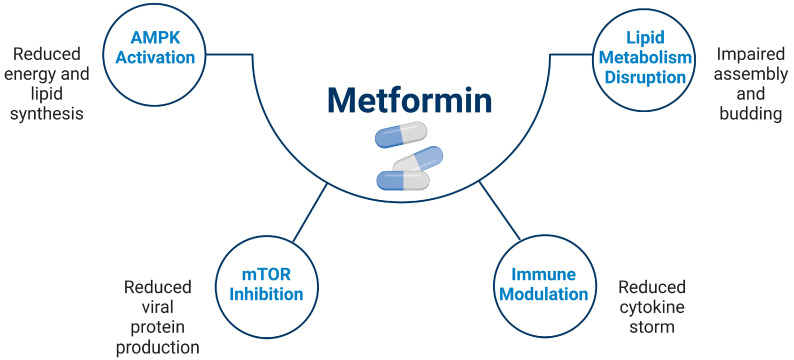
General antiviral mechanisms of metformin. This figure summarizes the general antiviral effects of metformin, which indirectly inhibit viral replication by targeting host cellular pathways. Metformin activates AMPK, reducing energy and lipid synthesis required for viral replication. It inhibits the mTOR pathway, limiting viral protein production, and disrupts lipid metabolism, impairing assembly and egress of viral particles. Metformin also downregulates host viral receptors, such as ACE2 (SARS-CoV-2), and modulates the immune response by reducing pro-inflammatory cytokines (e.g., IL-6, TNF-α) and suppressing immune activation, which decreases latent reservoirs (e.g., HIV).

Metformin, a first-line medication primarily prescribed for type 2 diabetes (T2DM), has garnered attention far beyond its glucose-lowering effects, showing remarkable potential in modulating host responses to viral and bacterial pathogens [1,2,3]. Initially celebrated for its ability to enhance insulin sensitivity and activate AMP-activated protein kinase (AMPK), metformin has demonstrated a breadth of influence on cellular metabolism, immune modulation, and oxidative stress, all mechanisms that extend its effects into the realm of infectious disease control [4,5,6] (Figure 1). As such, metformin’s influence on cellular processes has positioned it as a promising agent for enhancing host defenses against a range of viral infections and bacterial pathogens [7,8,9]. Some studies indicate metformin’s potential in enhancing therapeutic outcomes, with its effectiveness demonstrated in bacterial and cancer cell activity [2,10,11,12]. Metformin demonstrates pleiotropic benefits, contributing to the improvement of multiple conditions, including thyroid-related disorders and other diseases [13,14,15,16,17]. Metformin is associated with minimal side effects compared to other medications, with the most common being gastrointestinal disturbances, which are generally mild and transient [18,19,20,21,22].

**Figure 2:**
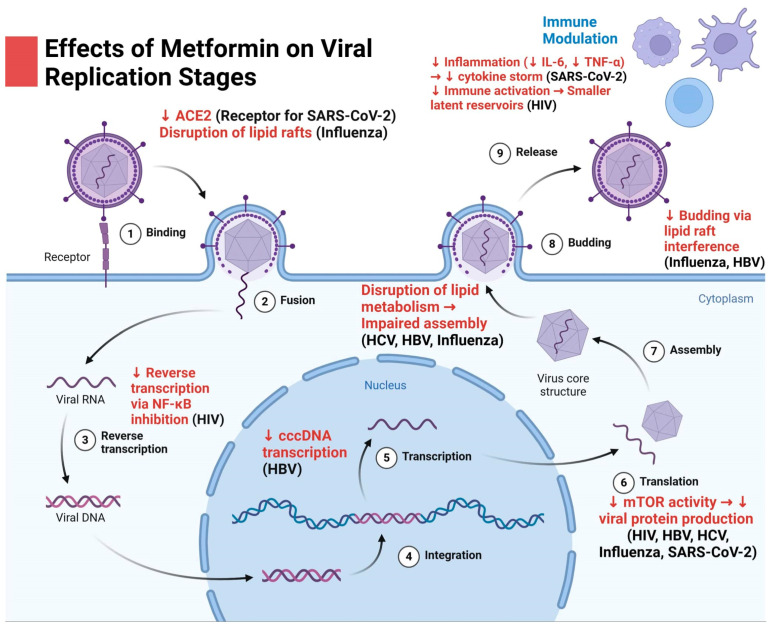
This schematic illustrates the antiviral effects of metformin across different stages of the viral replication cycle for SARS-CoV-2, HIV, HCV, HBV, and influenza. Metformin inhibits viral entry by downregulating ACE2 receptor expression (SARS-CoV-2) and disrupting lipid rafts (influenza). It suppresses genome replication by inhibiting viral polymerase activity (influenza, HCV), cccDNA transcription (HBV), and reverse transcription through NF-κB inhibition (HIV). Metformin reduces viral protein synthesis by inhibiting the mTOR pathway, affecting multiple viruses, including SARS-CoV-2. It impairs viral assembly and maturation by disrupting lipid metabolism (HCV, HBV, influenza) and inhibits viral egress by interfering with lipid-mediated budding (influenza, HBV). Additionally, metformin modulates the immune response by reducing inflammation and cytokine levels (e.g., IL-6 and TNF-α), mitigating cytokine storms (SARS-CoV-2), and decreasing immune activation to shrink latent reservoirs (HIV). Figure was designed using BioRender.com.

In the context of viral infections, metformin has shown the potential to interfere with viral replication and spread by creating an intracellular environment that is less conducive to viral survival [23,24] (Figure 2). One of the primary mechanisms underlying this antiviral effect is metformin’s activation of AMPK, which shifts host cellular metabolism away from the high-energy states that many viruses exploit for their replication cycles [25,26]. Additionally, metformin enhances the production of interferons and other cytokines, such as interleukin-6 (IL-6) and tumor necrosis factor-alpha (TNF-α), which play essential roles in the innate immune response to viral infection and chronic diseases [27,28,29]. Metformin’s influence on IL-6 and TNF-α production is context-dependent, varying with the inflammatory state and immune activation. In metabolic disorders such as type 2 diabetes mellitus, metformin activates AMPK, leading to NF-κB suppression and a subsequent reduction in cytokine levels [30,31]. Conversely, during immune responses, such as those triggered by infections, metformin may enhance cytokine production through MAVS and interferon signaling pathways [32,33]. These context-specific effects underscore the complex role of metformin as an immunomodulator rather than a straightforward inhibitor or stimulator of cytokine activity [34,35]. Through these pathways, metformin can help mount a more robust immune response, contributing to the containment of various viruses, including influenza, SARS-CoV-2, and cytomegalovirus [36,37,38].

Metformin’s modulation of inflammatory responses provides a dual benefit by enhancing immune cell recruitment to infection sites while preventing excessive inflammation that can cause tissue damage [27,39,40,41]. This balanced immune response can improve outcomes, especially in chronic infections or viral infections that might lead to significant tissue damage [42,43,44,45]. These combined mechanisms suggest that metformin’s effects extend beyond glucose regulation, impacting pathways critical for antiviral defenses [46,47,48,49].

## 2. Metformin in Viral Infections and Its Therapeutic Applications Across Multiple Pathogens

### 2.1. Metformin’s Antiviral Potential Against Influenza: Mechanisms and Therapeutic Insight

Influenza viruses, precisely types A and B, are segmented RNA viruses belonging to *the Orthomyxoviridae* family, which utilize host cell machinery to replicate and undergo frequent antigenic variation, contributing to seasonal epidemics and occasional pandemics [50,51]. Metformin has garnered significant attention for its potential antiviral effects, particularly against the influenza virus [52,53] (Table 1). Emerging research has highlighted the drug’s ability to modulate host cellular pathways, inhibiting viral replication [54,55]. The primary mechanism of metformin’s antiviral action involves the activation of AMPK, a central regulator of cellular energy homeostasis [56,57]. By activating AMPK, metformin disrupts metabolic processes essential for viral replication, thereby hindering the influenza virus’s ability to use host resources efficiently [38,58]. This metabolic interference results in a cellular environment less conducive to viral propagation [54,59].

In addition to altering metabolic pathways, metformin exerts significant immunomodulatory effects [65,66]. The activation of AMPK leads to the downregulation of pro-inflammatory cytokines, such as IL-6 and TNF-α, which are typically elevated during influenza infections and contribute to severe inflammatory responses [60,67,68]. Metformin may help mitigate hyperinflammation-related complications, such as acute respiratory distress syndrome (ARDS), by tempering the inflammatory cascade [69,70]. Furthermore, metformin appears to influence the innate immune response by enhancing the efficiency of autophagy, a process critical for the clearance of viral particles and damaged cellular components [71,72]. Enhanced autophagy facilitates viral elimination and preserves cellular integrity during infection [73,74].

Experimental evidence from in vitro studies has shown that metformin treatment significantly reduces viral loads within infected cell cultures [75,76]. This observation suggests a potential direct impact on virus–host interactions, possibly through the inhibition of viral entry or the disruption of viral genome replication [77,78]. Animal models infected with the influenza virus have also demonstrated the protective effects of metformin, with treated subjects displaying lower viral titers, reduced lung inflammation, and improved survival outcomes compared to untreated controls [79,80]. The immunomodulatory role of metformin may be particularly beneficial in moderating the host’s inflammatory response, thus preventing tissue damage caused by excessive immune activation [46,81].

Metformin influences the expression of several key genes involved in the antiviral response to influenza. It activates the AMPK gene, which plays a central role in cellular energy regulation and restricts viral replication by reducing available energy sources [82,83]. Metformin also upregulates interferon-beta 1 (IFNB1), which enhances the antiviral immune response by promoting interferon production [84]. The drug further stimulates microtubule-associated protein 1 light chain 3 (LC3) and Beclin-1 (BECN1) genes, essential for autophagy processes that help degrade intracellular influenza particles [85]. Metformin modulates superoxide dismutase 2 (SOD2), increasing reactive oxygen species that can directly damage viral structures [86,87,88].

Metformin has been shown to interfere with vaccine-induced immune responses in specific contexts [38]. It was reported that metformin negated the trained immunity induced by the mucosal vaccine MV130, reducing its protective effects against viral respiratory infections [56]. Similarly, metformin impaired antibody responses and IFN-α expression following influenza vaccination in T2DM patients, potentially compromising long-term immunity [63].

Moreover, metformin has been reported to improve the efficacy of conventional antiviral treatments when administered concurrently [63,89,90]. This synergistic effect may be due to its ability to enhance the overall antiviral response while reducing inflammation [61,91,92]. Some observational studies in humans have indicated that individuals with T2DM who are on metformin therapy tend to experience less severe influenza symptoms and lower hospitalization rates compared to those not receiving the medication [38,62]. Despite these promising findings, the exact molecular mechanisms underlying metformin’s antiviral effects remain a subject of ongoing research [93,94]. The potential roles of AMPK-mediated inhibition of mTOR signaling pathways, modulation of cellular redox status, and alteration of lipid metabolism are areas of particular interest [64,95].

Additionally, metformin may indirectly affect viral replication through its influence on mitochondrial function and the reduction of oxidative stress, further contributing to an inhospitable environment for the virus [96]. The drug’s capacity to improve mitochondrial biogenesis and reduce reactive oxygen species (ROS) production has implications for cellular health and viral control [97,98]. Understanding the multifaceted impact of metformin on cellular and viral processes could provide insights into novel therapeutic approaches [46,99]. Thus, while the evidence supports metformin’s potential as a complementary antiviral agent, comprehensive clinical trials are required to establish its efficacy and safety for widespread use in treating influenza infections, particularly in vulnerable and immunocompromised populations [38,100].

### 2.2. Metformin in the Context of COVID-19: Mechanisms of Action and Its Potential as a Therapeutic Agent Against SARS-CoV-2

SARS-CoV-2, the causative agent of the COVID-19 pandemic, has prompted extensive research into potential therapeutic agents, including metformin, which may modulate host immune responses and alter the disease trajectory through various cellular mechanisms [101,102,103,104,105]. Metformin has attracted attention for its potential impact on SARS-CoV-2 infection and the progression of COVID-19 [46,106,107] (Table 2). The drug’s anti-inflammatory and immunomodulatory effects are particularly relevant, given the role of excessive inflammation, or cytokine storm, in severe cases of COVID-19 and chronic diseases [108,109,110]. A central mechanism through which metformin may exert its protective influence is the activation of AMPK, which is linked to the inhibition of the mTOR pathway [111,112]. This pathway is involved in immune cell activation and inflammatory responses, and its modulation by metformin could help dampen hyperinflammation and promote more balanced immune regulation during SARS-CoV-2 infection [101,113,114].

Metformin also reduces oxidative stress, which is significantly elevated in severe COVID-19 cases [136,137]. By decreasing the production of ROS and enhancing mitochondrial function, metformin may mitigate cellular damage caused by viral infection and excessive inflammation [123,138]. Observational studies have indicated that diabetic patients using metformin prior to contracting SARS-CoV-2 have a lower risk of severe complications and mortality compared to those not on metformin therapy [118,124,131,139]. Nevertheless, these results are preliminary, and some studies, including those examining hematological markers, are necessary to establish a definitive causal relationship [121,122,140].

Metformin’s effects on the renin–angiotensin–aldosterone system (RAAS) have also been considered necessary, as SARS-CoV-2 utilizes the angiotensin-converting enzyme 2 (ACE2) receptor for cell entry [102,115,141]. Although the exact relationship between metformin and ACE2 expression is not fully understood, it has been hypothesized that the drug might modulate ACE2 levels, potentially affecting viral entry or disease severity [24,115,142,143]. Moreover, metformin improves endothelial function, which may offer protection against vascular complications commonly observed in COVID-19, such as endothelial damage, elevated blood pressure, and thrombosis [47,126,130,144,145,146].

The influence of metformin on gut microbiota is another area of interest, given the role of gut health in immune function and systemic inflammation [117,125,127,147,148,149]. Metformin could potentially reduce systemic inflammation and enhance immune resilience by altering microbial composition and promoting gut barrier integrity [23,119,122,150]. Preliminary studies suggest that metformin and other medications might also inhibit viral replication, although the direct anti-viral activity against SARS-CoV-2 remains to be validated [26,120,128,129,151,152,153].

Another mechanism through which metformin may benefit COVID-19 patients is by improving glucose metabolism and reducing insulin resistance [116,154,155]. Since hyperglycemia and insulin resistance are associated with poorer outcomes in COVID-19, metformin’s glucose-lowering properties could play a role in better disease management [156,157]. The idea of re-purposing metformin as an adjunctive treatment for COVID-19 is under active investigation, especially for high-risk groups, such as individuals with diabetes or obesity [118].

### 2.3. Metformin and HIV: Exploring Its Potential in Modulating Immune Responses and Enhancing Treatment Outcomes

Human Immunodeficiency Virus (HIV), a virus that causes chronic immune system dysfunction, remains a significant global health challenge, and recent research has explored the potential benefits of repurposing existing medications, such as metformin, to improve outcomes in people living with HIV [94,158,159]. Metformin has shown promise in modulating immune responses and could influence the course of HIV infection [158,160] (Table 3). Emerging evidence suggests that metformin may have immunoregulatory and anti-inflammatory properties relevant to HIV-induced chronic immune activation [158,161]. One of the critical mechanisms by which metformin exerts its effects is activating AMPK, a crucial regulator of cellular metabolism. AMPK activation by metformin has been associated with inhibiting the mTOR signaling pathway, which is involved in T-cell activation and proliferation [162,163]. By modulating these pathways, metformin may help to reduce the hyperactivation of immune cells that is characteristic of chronic HIV infection [164,165].

Furthermore, metformin has been observed to influence the function of regulatory T cells (Tregs), which play a vital role in maintaining immune homeostasis [173,174]. Enhancing Treg activity could potentially mitigate the immune dysregulation seen in people living with HIV [175,176]. Studies have also indicated that metformin may decrease systemic inflammation, as evidenced by reduced levels of inflammatory markers such as IL-6 and C-reactive protein (CRP) [177,178]. This reduction in inflammation may be beneficial in managing HIV-associated comorbidities, such as cardiovascular disease and metabolic syndrome, which are exacerbated by chronic inflammation [179,180].

Metformin’s impact on gut microbiota has garnered interest, as dysbiosis is a known factor in the immune dysfunction observed in HIV [181,182,183]. By improving gut barrier integrity and altering microbial composition, metformin may help reduce microbial translocation, a driver of systemic inflammation in HIV [184,185,186,187]. Studies have shown that metformin can reduce HIV replication in specific cell models, although the clinical significance of this finding remains unclear [,^188^,^189^]. The potential of metformin to complement antiretroviral therapy (ART) is an area of ongoing research, as it may enhance immune recovery and reduce residual inflammation despite effective viral suppression [94,158].

The studies collectively highlight the intricate interplay between antiretroviral therapies (ART) and metabolic health in people living with HIV [166,172]. Maraviroc (MVC), metformin, or their combination did not significantly reduce liver fat compared to antiretroviral therapy (ART) alone [166]. In contrast, metformin increased HIV transcription through CREB phosphorylation, suggesting potential metabolic interactions.. Combining metformin with dolutegravir, noting an increase in blood glucose levels [172].

Observational data have indicated that people with HIV who use metformin for diabetes management may experience slower progression of HIV-associated complications [190,191]. Nonetheless, despite the substantial body of research in this field, randomized clinical trials are required to validate these effects and to establish the optimal dosing and safety profile in the context of HIV [192,193,194]. The immunometabolic effects of metformin make it a candidate for further exploration as part of a comprehensive strategy to address the long-term health challenges faced by people living with HIV [195,196].

### 2.4. Metformin in Hepatitis C: Potential Therapeutic Effects on Viral Replication, Inflammation, and Hepatic Fibrosis

Hepatitis C virus (HCV), a hepatotropic, single-stranded RNA virus of the *Flaviviridae* family, is a primary global health concern due to its ability to cause chronic liver disease, leading to cirrhosis, hepatocellular carcinoma (HCC) and increased mortality rates, particularly in individuals with comorbid conditions such as insulin resistance, metabolic syndrome, and HIV coinfection [197,198,199]. Metformin has gained attention for its potential impact on the pathogenesis of chronic HCV infection, offering a promising adjunctive approach to managing this viral disease (Table 4). HCV infection is characterized by persistent liver inflammation, immune dysregulation, and the progressive development of fibrosis, which can eventually lead to cirrhosis and HCC [200,201]. The potential benefits of metformin in HCV infection are thought to stem from its ability to modulate several key cellular pathways involved in viral replication, inflammation, and hepatic fibrosis [202,203]. One of the primary mechanisms by which metformin exerts its effects is activating AMPK, a crucial regulator of cellular energy homeostasis [163,204].

AMPK is activated by metformin and inhibits the mTOR signaling pathway, a critical regulator of cell growth, protein synthesis, and immune cell activation [162,216]. This inhibition of mTOR may attenuate the inflammatory response central to the chronic liver injury observed in HCV infection [217,218]. In addition to its effects on mTOR, AMPK activation by metformin has been shown to reduce oxidative stress, a hallmark of HCV-induced liver damage [219,220]. ROS, produced during viral replication and inflammation, contribute to hepatocellular injury, fibrosis, and liver disease progression [221,222]. By reducing ROS production, metformin may help protect against cellular damage and limit the advancement of fibrosis [223,224].

Moreover, metformin’s ability to enhance insulin sensitivity and reduce hyperglycemia is particularly relevant in HCV patients who exhibit insulin resistance [225,226]. This condition exacerbates liver damage and accelerates disease progression. Insulin resistance in the context of HCV infection is associated with an increased risk of steatosis, advanced fibrosis, and poor treatment outcomes [227,228]. By improving insulin sensitivity, metformin may help mitigate these metabolic disturbances and thereby slow the progression of liver disease [229]. Additionally, metformin has been reported to exert an anti-inflammatory effect by decreasing the levels of pro-inflammatory cytokines such as IL-6 and TNF-α, which are elevated in chronic HCV infection and contribute to hepatic inflammation and fibrosis [36,230].

The interplay between metformin and immune modulation in HCV infection is also interesting [226,231]. Metformin may influence the function of innate immune cells, such as macrophages and dendritic cells, by reducing their activation and promoting a more balanced immune response [40,232]. This modulation of the immune system could help reduce the chronic inflammation and immune-mediated liver damage characteristic of HCV infection [233]. Additionally, metformin has been shown to inhibit the activation of hepatic stellate cells (HSCs), which play a crucial role in developing liver fibrosis [234,235]. By inhibiting HSCs activation and collagen deposition, metformin may help prevent or slow the progression of fibrosis in HCV-infected individuals [236].

Studies have suggested that metformin may also directly impact HCV replication [205]. While the exact mechanisms are not fully understood, some evidence points to the potential viral entry or replication inhibition within hepatocytes [237]. The potential for metformin to complement antiviral therapies in HCV infection is an area of ongoing research, particularly in individuals with comorbidities such as diabetes or metabolic syndrome [238,239]. Given that insulin resistance is a known risk factor for HCV-related liver disease, metformin’s effects on glucose metabolism and its potential to improve hepatic lipid metabolism are additional benefits in this context [240].

Furthermore, metformin’s effects on the gut microbiota have gained attention in the context of liver disease [241,242]. Dysbiosis, or an imbalance in the gut microbiome, has been implicated in the pathogenesis of liver diseases, including HCV infection [243,244]. Metformin has been shown to modulate gut microbiota composition, which could lead to reduced intestinal permeability and decreased microbial translocation, thereby lowering systemic inflammation and its impact on the liver [245,246]. By improving gut barrier function, metformin may reduce the chronic low-grade inflammation observed in HCV patients [247,248].

While the available preclinical data support metformin’s potential as a therapeutic adjunct in HCV infection, clinical evidence remains limited, and more randomized controlled trials are needed to understand its efficacy and safety profile in this context fully [11,200,249]. Combining metformin with direct-acting antiviral agents (DAAs) for HCV may represent an innovative therapeutic strategy, potentially enhancing viral eradication and improving liver function [207,250]. Metformin shows potential in modulating antiviral therapy for hepatitis C, especially when combined with sofosbuvir, velpatasvir, and ledipasvir [202,251]. Co-administration of metformin with DAAs like ledipasvir and sofosbuvir may offer a pathophysiological advantage in managing patients with hepatitis C and concurrent metabolic disorders [250,252]. However, the effects of metformin on HCV treatment outcomes are yet to be conclusively demonstrated in clinical trials [253,254].

The studies provide valuable insights into the role of metformin in managing hepatocellular carcinoma (HCC) [205,213]. A significant reduction in HCC risk was demonstrated with metformin use in patients with diabetes and chronic hepatitis C after antiviral therapy [205]. In contrast, metformin, along with statins and aspirin, did not show a statistically significant association with improved outcomes in liver transplant recipients with HCC [213].

### 2.5. Metformin in Hepatitis B: Targeting Insulin Resistance, Inflammation, and Fibrosis in Chronic Liver Disease

Hepatitis B virus (HBV) is a major global health threat, with chronic infection potentially leading to cirrhosis, HCC, and significant morbidity and mortality [255]. While antiviral therapies, such as nucleos(t)ide analogs, are widely used to suppress HBV replication, there is growing interest in repurposing existing medications to improve treatment outcomes and address co-morbidities associated with HBV infection [256]. Metformin has garnered attention due to its pleiotropic effects and potential impact on HBV pathogenesis [13,257] (Table 4). Chronic HBV infection is often associated with dysregulated immune responses, inflammation, and liver damage, and metformin may influence several key processes that contribute to these disease mechanisms [258,259].

Insulin resistance, a common comorbidity in HBV patients, particularly in those with metabolic syndrome or non-alcoholic fatty liver disease (NAFLD), is known to exacerbate liver damage and increase the risk of fibrosis [260,261]. Metformin’s ability to improve insulin sensitivity and reduce hyperglycemia is particularly relevant in HBV-infected individuals, as insulin resistance is associated with worse outcomes in chronic liver disease [262,263]. Metformin may reduce the risk of steatosis, hepatic inflammation, and fibrosis progression in patients with HBV infection by improving glucose metabolism [236,264]. Moreover, metformin may influence immune modulation in HBV infection by affecting the function of innate and adaptive immune cells [257]. Metformin has been demonstrated to regulate macrophage activation, promoting an anti-inflammatory phenotype, which could help reduce the chronic inflammation observed in HBV-related liver disease [265,266]. The modulation of T-cell responses is also interesting, as HBV infection often leads to a dysregulated immune response characterized by persistent viral replication and immune exhaustion [267,268]. Metformin may promote a more balanced immune response by modulating the immune system, which could contribute to viral control and limit hepatic damage [38,269].

The potential role of metformin in mitigating HBV-related liver fibrosis is particularly compelling. HSCs, which are responsible for producing extracellular matrix proteins and fibrosis, are activated in response to chronic inflammation and viral replication [270,271]. Studies have shown that metformin can inhibit the activation of HSCs, thereby reducing collagen deposition and fibrosis progression [272]. By targeting this essential aspect of the fibrotic process, metformin may slow the progression to cirrhosis and hepatocellular carcinoma, the most severe outcome of chronic HBV infection [273,274].

Furthermore, metformin’s effect on gut microbiota composition may influence HBV pathogenesis, as gut dysbiosis and microbial translocation have been implicated in the progression of liver disease [275,276]. Metformin has been shown to alter the gut microbiome, potentially improving gut barrier function and reducing systemic inflammation [277,278]. This could benefit liver inflammation and fibrosis, as the gut-liver axis plays a significant role in the pathogenesis of chronic liver diseases, including HBV [279].

Clinical studies examining the effects of metformin in HBV-infected individuals are limited but suggest that metformin use may be associated with improved liver function and reduced levels of inflammatory markers [240,280]. Observational data indicate that patients with diabetes and chronic HBV who are treated with metformin may experience better liver outcomes compared to those not using the drug [281,282]. The use of metformin in HBV infection could also help manage comorbid conditions, such as diabetes and NAFLD, which are prevalent in HBV patients and complicate treatment and disease progression [283].

### 2.6. Metformin as an Antiviral: Potential Applications Against Cytomegalovirus, Herpes Simplex Virus, Zika Virus, Dengue Virus, Epstein–Barr Virus, Human Papillomavirus, and Others

Evidence suggests that metformin may inhibit the replication of several viruses, including cytomegalovirus (CMV), herpes simplex virus (HSV), Zika virus, dengue virus, Epstein–Barr virus (EBV), and human papillomavirus (HPV) [36,284,285] (Table 5). The mechanism of action appears to involve its ability to modulate cellular pathways that are critical for viral replication [94,286].

Studies show that metformin can reduce viral loads in CMV, a virus that causes chronic infection in immunocompromised individuals [288]. The drug downregulates the mammalian target of the mTOR pathway, which is essential for CMV’s protein synthesis and replication processes [285,303]. Metformin may limit CMV replication by inhibiting mTOR, suggesting its potential in therapeutic strategies against CMV-induced diseases [304,305]. CMV-induced “inflammaging” from immune cell senescence can be mitigated with interventions like senolytics or metformin, enhancing immune function in older adults [306]. CMV replication depends on mitochondrial function, with metformin showing promise as a repurposed antiviral due to its inhibition of viral replication [288]. Metformin restores impaired CD8⁺ T-cell functionality in diabetic patients, linking metabolic improvement to enhanced viral immunity and reduced susceptibility to CMV [289].

Similarly, metformin has shown inhibitory effects on HSV replication, another virus known for establishing persistent infections. HSV relies on cellular resources to synthesize viral proteins and assemble virions [307,308]. Metformin’s activation of AMPK restricts these processes, potentially leading to reduced HSV reactivation rates in latently infected individuals [36,309]. Metformin alleviates herpetic stromal keratitis severity while preserving immune function, suggesting it as a safer alternative compared to 2-deoxy-D-glucose [291].

Regarding arboviruses such as Zika and dengue, metformin interferes with viral replication by modulating oxidative stress levels in host cells [284]. These viruses generate ROS for efficient replication [284,310]. Metformin, known for its antioxidant properties, reduces ROS production, indirectly impairing the replication efficiency of these viruses [311]. This effect on oxidative stress has made metformin a candidate for adjunctive therapy in treating Zika and dengue infections, which are often complicated by inflammation and tissue damage [295,312]. Metformin exhibited poor anti-DENV activity in vitro, with pro-DENV effects observed in certain cell lines, and in vivo administration did not reduce viral titers or improve disease severity [295]. These findings highlight the need for cautious consideration of metformin use in managing dengue virus infection, especially at high doses [313].

In the context of EBV, metformin may impact its latent and lytic phases [314]. EBV-associated diseases are often triggered by the virus’s reactivation from latency, which requires a shift in host cellular metabolism [315,316]. Metformin’s ability to activate AMPK and inhibit mTOR has been linked to inhibiting EBV lytic reactivation, thus limiting its pathogenic potential [317,318].

Metformin may also reduce replication rates of the HPV by influencing cellular differentiation and proliferation processes, which HPV relies on to complete its life cycle [319]. By regulating these cellular pathways, metformin could impair HPV replication, thus reducing the risk of HPV-related cancers [320,321]. T-cell spatial distribution in HPV-positive tumors predicts response to immunotherapies, with metformin modulating CD8⁺ T-cell densities in treated cancers [302].

In addition to its metabolic effects, metformin exhibits immunomodulatory properties that may enhance the body’s antiviral defenses [322]. Research also indicates that metformin can inhibit adenovirus replication by reducing the expression of E1A proteins, essential for viral DNA replication and cell lysis [323,324,325]. This mechanism might particularly interest immunocompromised patients, where adenovirus infections are frequently more severe [326].

Moreover, studies have explored metformin’s role in controlling poxvirus infections, particularly vaccinia virus, where its influence on cellular autophagy appears relevant [327,328]. Metformin could impair viral assembly and release by modulating autophagy, thereby inhibiting the spread of infection [26,164].

## 3. Conclusions

Metformin, widely recognized for its anti-diabetic properties, demonstrates significant potential as a broad-spectrum antiviral agent due to its modulation of critical cellular pathways that impact viral replication. Beyond its effects on influenza, HIV, SARS-CoV-2 (COVID-19), HCV, and HBV, metformin shows inhibitory effects on other viruses, including cytomegalovirus (CMV), herpes simplex virus (HSV), Zika virus, dengue virus, Epstein–Barr virus (EBV), and human papillomavirus (HPV). Metformin’s activation of AMPK limits viral replication by reducing cellular energy resources, to the detriment of viruses like influenza and SARS-CoV-2, and potentially mitigating cytokine storms in COVID-19. It also inhibits the mTOR pathway in HIV, reducing viral protein synthesis and chronic inflammation. For HBV and HCV, metformin disrupts viral replication and host metabolism, improving liver function and potentially lowering viral load. Metformin’s activation of AMPK reduces cellular energy resources that viruses typically exploit, limiting replication for various viruses. Additionally, its inhibition of the mTOR pathway—crucial for processes like protein synthesis in CMV and reactivation in EBV—further suppresses viral activity.

Metformin’s modulation of oxidative stress and ROS levels provides an antiviral effect for arboviruses like Zika and dengue, as these viruses rely on elevated ROS for replication. Its ability to regulate cellular differentiation and immune response may also interfere with HPV’s life cycle. Collectively, these multifaceted actions highlight metformin’s potential as adjunctive therapy for a broad range of pathogens, opening promising avenues for future host-targeted treatments.

## Figures and Tables

**Table 1:** Summary of studies investigating impact of metformin on influenza virus.

Author and Year	Type of Study	Key Findings
Fu-Shun Yen et al., 2022 [52]	Cohort Study	Pre-influenza vaccination metformin use in older adults with T2DM significantly reduced the risks of severe influenza-related complications and mortality, with greater benefits observed with longer usage.
Han Sol Lee et al., 2023 [23]	Experimental and Statistical Analysis	Metformin reduced influenza A virus-related cardiovascular risks by inhibiting viral replication and cytokine expression (MCP-1, IP-10) through AKT/MAPK signaling regulation.
Dominique E. Martin et al., 2023 [58]	Pilot Double-Blinded Placebo-Controlled Trial	Metformin may enhance immune resilience in older adults by improving specific flu vaccine responses and reducing markers of T-cell exhaustion.
Elizabeth Greene et al., 2024 [54]	Retrospective Observational Study	Metformin use in diabetic patients significantly reduces the likelihood of hospitalization following an emergency department visit for influenza.
Tammy H. Cummings et al., 2022 [53]	Retrospective Cohort Study	Metformin use is associated with reduced influenza mortality in patients with obesity, likely due to its effects on T-cell function and immune response.
Paola Brandi et al., 2022 [56]	Experimental Study (Mouse Model)	The inactivated mucosal vaccine MV130 induces trained immunity, offering protection against viral respiratory infections, but this protection is negated by metformin.
Robert E. Brown et al., 2022 [60]	Case Study with Morphoproteomics	Morphoproteomic analysis suggests metformin and vitamin D3 could serve as adjunctive therapies to improve immune response and prevent severe outcomes in pulmonary H1N1 influenza.
Daniela Frasca et al., 2021 [61]	Experimental Study	Metformin improves B-cell function and enhances antibody responses in elderly individuals with T2DM, supporting its potential as an anti-aging agent for immune function.
Wen-Rui Hao et al., 2023 [62]	Retrospective Study	Influenza vaccination reduces the risk of chronic kidney disease and the need for dialysis in patients with hypertension, with a dose-dependent protective effect observed across both influenza and non-influenza seasons.
Wipawee Saenwongsa et al., 2020 [63]	Observational Study	Metformin treatment in T2DM impairs the antibody response and interferon-alpha (IFN-α) expression following seasonal influenza vaccination, potentially hindering long-term protection. This finding suggests that the standard influenza vaccine may not be fully effective for T2DM patients and highlights the need for improved vaccine strategies for this group.
Aimin Yang et al., 2021 [64]	Cohort Study (Registry-based)	Long-term metformin use in T2DM individuals is associated with a lower risk of pneumonia hospitalisation and related mortality.

**Table 2:** Summary of studies investigating the impact of metformin on SARS-CoV-2 virus.

Author and Year	Type of Study	Key Findings
Malhotra et al., 2020 [115]	Preclinical/Clinical	Metformin may enhance ACE2 expression, potentially offering cardiopulmonary protection in COVID-19 by regulating the renin–angiotensin–aldosterone system (RAAS).
Bramante et al., 2022 [116]	Randomized, Placebo-Controlled Trial	No significant reduction in primary composite endpoint (hypoxemia, ED visit, hospitalization, or death) for metformin (OR 0.84, *p* = 0.19), ivermectin (OR 1.05, *p* = 0.78), or fluvoxamine (OR 0.94, *p* = 0.75). Secondary analysis showed metformin reduced ED visits, hospitalization, or death (OR 0.58, *p* = 0.02), but not significantly for ivermectin or fluvoxamine.
Pavlo Petakh et al., 2023 [117]	Observational Study	COVID-19 patients with T2DM have reduced gut microbiota alpha-diversity.
Jean-Daniel Lalau, Abdallah Al-Salameh, Samy Hadjadj, et al., 2021 [118]	Observational Study	Metformin use in patients with T2DM hospitalized for COVID-19 was associated with a lower 28-day mortality rate (16.0% vs. 28.6%, *p* < 0.0001) and reduced odds of death (OR 0.710, 95% CI [0.537−0.938]) compared to non-users.
Pavlo Petakh et al., 2024 [119]	Single-center Prospective Observational Study	Metformin therapy was associated with reduced expression of key genes (PRKAA1, SLC2A1, MTOR) involved in Th1/Th17 cell differentiation and inflammatory pathways.
Carolyn T Bramante et al., 2023 [113]	Randomised Phase 3 Trial	Metformin reduced long COVID incidence by 41% compared to placebo, with the greatest effect when started early.
Carolyn T Bramante et al., 2024 [120]	Randomised Clinical Trial	Metformin reduced SARS-CoV-2 viral load by 3.6-fold, hospitalizations by 58%, and long COVID by 42%.
David R Boulware et al., 2023 [121]	Secondary Analysis of RCT Data	Vaccine-boosted participants experienced the least severe and shortest-lasting COVID-19 symptoms (*p* < 0.001).
Pavlo Petakh et al., 2022 [122]	Retrospective Study	COVID-19 patients with T2DM who used metformin before hospitalization had significantly lower CRP levels, suggesting anti-inflammatory benefits
Claudia Ventura-López et al., 2022 [24]	In vitro study & Phase IIb RCT	Metformin glycinate inhibited viral replication in vitro without cytotoxicity and reduced viral load and oxygen needs in vivo.
Fabio Petrelli et al., 2023 [123]	Meta-Analysis	Metformin use in diabetic patients with COVID-19 reduced the risk of severity, complications, and mortality compared to other treatments.
Giovanni Antonio Silverii et al., 2024 [124]	Retrospective Study	Metformin use was associated with a reduction in in-hospital mortality in people with diabetes, but the effect did not persist after adjusting for confounding factors using the COVID-19 Mortality Risk Score.
Pavlo Petakh et al., 2023 [125]	Observational Study	The *Firmicutes*/*Bacteroidetes* (F/B) ratio in gut microbiota was higher in patients with both T2D and COVID-19. F/B ratio positively correlated with CRP levels, and metformin treatment modified this relationship. The F/B ratio may serve as a biomarker for inflammation.
Verónica Miguel et al., 2023 [126]	Experimental Study	Metformin and baicalin enhanced fatty acid oxidation, improving mitochondrial function, reducing inflammation, fibrosis, and improving outcomes in COVID-19 patients and animal models with lung and kidney damage.
Pavlo Petakh et al., 2023 [127]	Observational Study	T2D patients with COVID-19 showed increased ***Clostridium*** and ***Candida***, and decreased ***Bifidobacterium*** and ***Lactobacillus***. Metformin use without antibiotics increased ***Bacteroides*** and ***Lactobacillus***, while decreasing ***Enterococcus*** and ***Clostridium***.
Yongwang Hou et al., 2024 [128]	Bioinformatics and Preclinical Study	Metformin may treat COVID-19/LUAD by regulating glucose metabolism and key signaling pathways like AMPK and mTOR, inhibiting cell proliferation.
H M Al-Kuraishy et al., 2023 [129]	Prospective Cohort Study	Metformin was more effective than other diabetic treatments in reducing inflammation, oxidative stress, and improving radiological and clinical outcomes in T2DM patients with COVID-19.
Pavlo Petakh et al., 2024 [130]	Observational Study	Metformin modulates T-cell mRNA expression: **FOXP3** (Treg marker) upregulated 1.96-fold, **RORC** (Th17 marker) downregulated 1.84-fold, and **TBX21** (Th1 marker) downregulated 11.4-fold. Patients not using metformin showed dysregulated immune profiles.
Muhilvannan Somasundaram et al., 2024 [131]	Retrospective Cohort Study	Metformin use was associated with shorter hospitalization, reduced mortality risk, and improved levels of LDH, CRP, and D-dimer in COVID-19 patients with diabetes.
Sky Qiu et al., 2024 [132]	Retrospective Cohort Study	Improved adherence to metformin (by 5% or 10%) was associated with a reduction in mortality risk from COVID-19, with a 1.26% absolute decrease in risk for a 10% adherence increase.
Thomas D Lockwood, 2024 [133]	Coordination Chemistry Analysis	Metformin and Zn^2+^ are suggested to have a mechanistic relationship in improving COVID-19 outcomes. Metformin enhances Zn^2+^ bioavailability and coordination, which may synergistically inhibit viral proteases and reduce inflammation, potentially improving outcomes when used together.
David C Harmon et al., 2024 [134]	Retrospective Cohort Study	Pre-admission metformin use was associated with reduced in-hospital mortality, lower risk of ICU admission, and less need for mechanical ventilation in hospitalized COVID-19 patients with diabetes. The effect was particularly notable in reducing mortality from respiratory causes.
Łukasz Lewandowski et al., 2024 [135]	Retrospective Cohort Study	Insulin and metformin showed weak associations with mortality, but their interactions with other treatments and factors like remdesivir, low-molecular-weight heparin, age, and hsCRP influenced death risk. RDW-SD was strongly associated with mortality, with a significant increase in death risk with higher RDW-SD.

**Table 3:** Effects of metformin in HIV-related studies.

Author and Year	Type of Study	Key Findings
Fert et al., 2024 [94]	Experimental study	Metformin decreased virion release, increased productively infected CD4lowHIV-p24+ T cells, enhanced tetherin and Bcl-2 expression, and improved recognition of infected cells by HIV-1 antibodies.
McCabe et al., 2024 [166]	Open-label, randomized trial	Neither maraviroc (MVC), metformin, nor their combination significantly reduced liver fat compared to ART alone in PWH with MAFLD.
Rezaei et al., 2024 [167]	Experimental study	Metformin increased HIV transcription, gene expression, and production via CREB phosphorylation and recruitment to the HIV LTR promoter.
McCrea et al., 2024 [168]	Phase 1, open-label study	Coadministration of islatravir with atorvastatin and metformin did not have a clinically meaningful effect on the pharmacokinetics of either drug.
Corley et al., 2024 [169]	Retrospective analysis, randomized and single-arm trials	Metformin reduced epigenetic age in monocytes but not in CD8+ T cells, suggesting cell-type-specific effects. Larger studies are needed to validate findings.
Nguyen et al., 2024 [170]	Physiologically based pharmacokinetic (PBPK) modeling study	Fostemsavir (a gp120-directed attachment inhibitor) and its active moiety temsavir showed no clinically relevant impact on metformin concentrations or inhibition of OCT1, OCT2, or MATE1/2K transporters. PBPK modeling confirmed no significant drug–drug interaction, supporting that no dose adjustment of metformin is required during coadministration with fostemsavir, despite initial in vitro data indicating potential transporter inhibition.
Mhlanga et al., 2024 [171]	Qualitative multi-method study	The study identified key interventions to reduce T2DM among older people living with HIV in Harare, including improved screening and health education. It also highlighted the use of metformin as a pharmacological intervention when lifestyle changes fail.
Hurbans et al., 2024 [172]	Prospective cohort study	Dolutegravir was generally safe and effective, but concomitant use of metformin led to increased blood glucose levels. Drug interactions were minimal, with only 0.7% of participants discontinuing dolutegravir due to interactions with supplements and antacids. Further investigation into dolutegravir-induced hyperglycemia is needed.

**Table 4:** Summary of studies on metformin and hepatitis C virus (HCV), chronic hepatitis B, and hepatocellular carcinoma (HCC) treatment outcomes.

Author and Year	Type of Study	Virus	Key Findings
Tsai et al., 2023 [205]	Cohort Study	HCV	Metformin significantly reduced HCC risk in patients with diabetes and chronic hepatitis C after successful antiviral therapy. The 5-year cumulative HCC incidence was 10.9% in non-metformin users vs. 2.6% in metformin users. A risk model identified cirrhosis and T2DM non-metformin use as the most critical factors for HCC prediction. Metformin also reduced liver-related complications.
Shimada et al., 2021 [206]	Cohort Study	HCV	Patients with high HbA1c (≥7.0%) had worse overall survival (55% vs. 71%) and relapse-free survival (13 vs. 26 months) in NBNC-HCC. High HbA1c was also associated with increased postoperative complications. Metformin use was linked to better survival and recurrence outcomes.
Lin et al., 2021 [207]	Experimental Study	HCV	Metformin inhibited Wnt/β-catenin signaling in chronic HCV-infected cells after DAA treatment, leading to decreased proliferation, increased apoptosis, and reversal of HCV-induced HCC.
Berk et al., 2020 [208]	Case Study	HCV	Successful treatment of HCV led to significant improvement in glycemic control in a patient with uncontrolled T2DM, with HbA1c dropping from 11.6% to 5.7% without any other interventions, suggesting potential benefits of HCV treatment on insulin sensitivity.
Abdel Monem et al., 2021 [209]	Randomized clinical trial	HCV	Metformin used in HCV-infected adolescents with beta thalassemia major led to significant improvement in oxidative stress markers, liver fibrosis, and liver enzyme levels, suggesting its potential as a hepatoprotective agent.
Valenti et al., 2022 [210]	Cohort Study	HCV	In patients treated with direct-acting antivirals for HCV, higher BMI and diabetes were linked to advanced fibrosis. Diabetes was also associated with poor liver stiffness improvement and increased risk of de novo HCC and cardiovascular events. Statin use was protective, and metformin showed a protective association against HCC.
Rodríguez-Escaja et al., 2021 [211]	Cohort Study	HCV	In patients with alcoholic or HCV cirrhosis, diabetes was not a risk factor for developing HCC. No significant differences in HCC incidence were found between diabetic and non-diabetic patients, even after adjusting for co-factors and excluding metformin use.
Thomaz et al., 2024 [212]	Clinical Pharmacology Study	HCV	Liver fibrosis stages affected the in vivo activity of organic cation transporters (OCT1/2) in HCV-infected patients. Advanced fibrosis and cirrhosis were associated with a 25% reduction in OCT1/2 activity after achieving sustained virologic response. No significant changes were observed in the early stages of treatment.
Chung et al., 2024 [213]	Retrospective Study	HBV	In a retrospective study of liver transplant recipients for HCC, statin, aspirin, and metformin use did not show a statistically significant association with improved HCC-related outcomes (recurrence or mortality). The study suggests no benefit for these drugs in post-LT HCC recurrence prevention, indicating the need for further prospective, multicenter studies to clarify any potential benefit.
Campbell et al., 2021 [214]	Meta-Analysis	HBV	This meta-analysis found that T2DM is a significant risk factor for HCC in individuals with chronic HBV infection, increasing the hazard of HCC by over 25%. The association was weakened in studies adjusted for metformin use, suggesting that further research on the impact of antidiabetic drugs and glycemic control is needed. Enhanced screening for HCC in individuals with HBV and diabetes is recommended.
Zhou et al., 2020 [215]	Experimental Study	HBV	CD39 and CD73 expression on B cells was reduced in chronic hepatitis B patients with high HBV DNA, HBeAg positivity, and active liver inflammation. This was linked to B-cell hyperactivation. Metformin reduced activation markers by regulating AMPK. Targeting the CD39/CD73/adenosine pathway using metformin could help reverse HBV-induced immune dysfunction.

**Table 5:** Summary of studies on effects of metformin and other interventions on viral infections.

Author and Year	Type of Study	Virus	Key Findings
Chen et al., 2022 [287]	Experimental study on mice and human myocardium	CMV	Bmi-1-RING1B prevents GATA4-dependent senescence-associated pathological cardiac hypertrophy (SA-PCH) by promoting selective autophagic degradation of GATA4. Autophagy activators like metformin or rapamycin may serve as therapeutic options to prevent SA-PCH and cardiac dysfunction.
Combs et al., 2021 [288]	In vitro experimental study	CMV	CMV replication depends on functional host mitochondria, and drugs targeting the electron transport chain, such as metformin, inhibit viral replication. Repurposing metformin as an antiviral is promising due to its established safety profile and ability to reduce CMV titers.
Nojima et al., 2020 [289]	Experimental study	CMV	T2DM impairs the multifunctionality of CD8⁺ PD-1⁺ T cells and links metabolic dysfunction to immune suppression. Metformin restores CD8⁺ T-cell function by enhancing glycolysis, improving cytokine production, and reducing tumor growth and viral susceptibility.
Poorghobadi et al., 2024 [290]	Mouse model experimental study	Herpes simplex virus 1 (HSV-1)	Ad-HSV-tk/GCV reduced tumor size and increased LC3B expression, promoting autophagy in multiple myeloma. Ad-IL-24 enhanced UPR gene expression but had a less pronounced effect on tumor reduction, and co-administration of Ad-HSV-tk and Ad-IL-24 showed no synergistic effect.
Berber & Rouse, 2022 [291]	Experimental study on HSV-1 in ocular infection	HSV-1	Metformin and 2-deoxy-d-glucose (2DG) reduced herpetic stromal keratitis (HSK) severity, but 2DG increased the risk of herpetic encephalitis due to enhanced HSV reactivation. Metformin was safer, maintaining inflammatory cell functionality, including IFN-γ-producing Th1 and CD8 T cells in the trigeminal ganglion.
Farfan-Morales et al., 2021 [284]	In vitro and in vivo studies on DENV and ZIKV	Dengue virus (DENV), Zika virus (ZIKV), yellow fever virus (YFV)	Metformin inhibited in vitro replication of DENV, ZIKV, and YFV, showing the strongest effect on DENV. MET reduced disease severity and increased survival in DENV-infected mice but failed to protect immunodeficient mice against ZIKV in vivo.
Wang et al., 2023 [292]	In vitro study on ZIKV infection in microglia	ZIKV	Metformin reduced ZIKV replication in microglia in a dose- and time-dependent manner. It modulated inflammatory responses, upregulating type I and III interferons (IFNα2, IFNβ1, IFNλ3) and downregulating ISGs like GBP4, OAS1, MX1, and ISG15. The findings suggest metformin may have therapeutic potential for ZIKV infection in microglia.
Singh et al., 2020 [293]	In vitro study on endothelial cells	ZIKV	The study explores how AMPK restricts ZIKV replication in endothelial cells. AMPK activation (via metformin or other activators) potentiates innate antiviral responses (e.g., IFNs, OAS2, ISG15) and inhibits glycolysis, which reduces viral replication. In contrast, inhibition of AMPK or increased glycolysis promoted virus replication.
Velazquez-Cervantes et al., 2024 [294]	In vitro study on trophoblast cell line	ZIKV	The study investigates the effects of metformin on ZIKV infection in a trophoblast cell line (JEG3). Metformin reduces viral replication and protein synthesis, reverses cytoskeletal changes, and reduces lipid droplet formation associated with the infection, suggesting metformin as a potential antiviral agent for ZIKV.
Cheang et al., 2021 [295]	In vitro and in vivo study	DENV	Metformin showed poor anti-DENV activity in vitro, with pro-DENV effects observed in certain cell lines (Vero cells). In vivo, oral administration of metformin did not reduce viral titers or improve disease severity in mouse models, and high doses worsened the outcome (higher viremia, mortality, and hyper-inflammation). The study suggests AMPK activation could be a potential host target.
Bonglack et al., 2021 [296]	In vitro study	Epstein–Barr virus (EBV)	EBV infection upregulates MCT1 and MCT4, supporting glycolysis. Dual inhibition of both transporters halts cell growth, causes lactate accumulation, decreases oxygen consumption, depletes glutathione, and enhances sensitivity to phenformin and metformin.
Hoppe-Seyler et al., 2021 [297]	In vitro study	Human papillomavirus (HPV)	Metformin downregulates E6/E7 oncogene expression in HPV-positive cervical and head/neck cancer cells through glucose and PI3K pathways. Despite E6/E7 repression, Metformin causes a reversible proliferative stop and prevents senescence induced by E6/E7 inhibition or chemotherapy, suggesting potential for repurposing metformin in cancer therapy.
Hsu et al., 2021 [298]	Nested case-control study	HPV	Metformin use was associated with a 56% lower likelihood of anal intraepithelial neoplasia (AIN) in type 2 diabetic patients. This suggests that metformin may offer protective effects against AIN, a precursor to anal cancer, potentially due to its influence on HPV-related pathways.
Veeramachaneni et al., 2021 [299]	Preclinical mouse model study	HPV	Long-term metformin treatment significantly reduced tumor growth, increased CD8+ T cells, and upregulated immune responses in head and neck cancer models. Acute metformin exposure, however, had limited antitumor effects. Combinatorial approaches with immune checkpoint inhibitors (ICIs) may enhance its therapeutic potential.
Wilkie et al., 2021 [300]	In vitro study on HPV-positive SCCHN	HPV	HPV-positive head and neck cancer cells exhibited a metabolically diverse phenotype. Sensitization to ionizing radiation (IR) required a combination of 2-deoxy-D-glucose and metformin, targeting both mitochondrial respiration and glycolysis. This approach could reduce radiation doses and minimize treatment impact on long-term function.
Sharma and Munger, 2020 [301]	In vitro study on HPV16 E7-expressing cells	Human papillomavirus 16 (HPV-16)	HPV16 E7 stabilizes the tumor suppressor TP53 via the long noncoding RNA (lncRNA) DINO, which is regulated by KDM6A. DINO levels increase in HPV16 E7-expressing cells and further stabilize TP53. Cells are sensitized to metabolic stress (e.g., by metformin) and chemotherapy (e.g., doxorubicin) in a DINO-dependent manner, linking DINO to TP53 activation and cell death response.
Curry et al., 2023 [302]	Clinical trial, analysis of tumor samples	HPV	After treatment with durvalumab and metformin, significant changes were observed in CD8+ and FoxP3+ T-cell densities and spatial distributions in head and neck squamous cell carcinoma (HNSCC). HPV-positive tumors had greater intercellular distances (ID) than HPV-negative ones. Pathologic responders showed higher CD8+ density and ID. These findings suggest that T-cell distribution patterns may predict response to immune checkpoint inhibitors.

## References

[1] Dutta S., Shah R.B., Singhal S., Dutta S.B., Bansal S., Sinha S., Haque M. (2023). Metformin: A Review of Potential Mechanism and Therapeutic Utility Beyond Diabetes. Drug Des. Dev. Ther..

[2] Amengual-Cladera E., Morla-Barcelo P.M., Morán-Costoya A., Sastre-Serra J., Pons D.G., Valle A., Roca P., Nadal-Serrano M. (2024). Metformin: From Diabetes to Cancer-Unveiling Molecular Mechanisms and Therapeutic Strategies. Biology.

[3] Redkva O.V., Babinets L.S., Halabitska I.M. (2021). Evaluation of Parameters of Actual Typical Pathogenetic Syndromes in Comorbidity of Type 2 Diabetes Mellitus and Chronic Pancreatitis. Wiad. Lek..

[4] Du M.R., Gao Q.Y., Liu C.L., Bai L.Y., Li T., Wei F.L. (2022). Exploring the Pharmacological Potential of Metformin for Neurodegenerative Diseases. Front. Aging Neurosci..

[5] Zhou G., Myers R., Li Y., Chen Y., Shen X., Fenyk-Melody J., Wu M., Ventre J., Doebber T., Fujii N. (2001). Role of AMP-activated protein kinase in mechanism of metformin action. J. Clin. Investig..

[6] Halabitska I., Petakh P., Kamyshna I., Oksenych V., Kainov D.E., Kamyshnyi O. (2024). The interplay of gut microbiota, obesity, and depression: Insights and interventions. Cell. Mol. Life Sci. CMLS.

[7] Xiao Y., Liu F., Li S., Jiang N., Yu C., Zhu X., Qin Y., Hui J., Meng L., Song C. (2020). Metformin promotes innate immunity through a conserved PMK-1/p38 MAPK pathway. Virulence.

[8] Rahman M.A., Sarker A., Ayaz M., Shatabdy A.R., Haque N., Jalouli M., Rahman M.D.H., Mou T.J., Dey S.K., Hoque Apu E. (2024). An Update on the Study of the Molecular Mechanisms Involved in Autophagy during Bacterial Pathogenesis. Biomedicines.

[9] Naicker N., Sigal A., Naidoo K. (2020). Metformin as Host-Directed Therapy for TB Treatment: Scoping Review. Front. Microbiol..

[10] Bilyi A.K., Antypenko L.M., Ivchuk V.V., Kamyshnyi O.M., Polishchuk N.M., Kovalenko S.I. (2015). 2-Heteroaryl-[1,2,4]triazolo[1,5-c]quinazoline-5(6 H)-thiones and Their S-Substituted Derivatives: Synthesis, Spectroscopic Data, and Biological Activity. ChemPlusChem.

[11] Galal M.A., Al-Rimawi M., Hajeer A., Dahman H., Alouch S., Aljada A. (2024). Metformin: A Dual-Role Player in Cancer Treatment and Prevention. Int. J. Mol. Sci..

[12] Nosulenko I.S., Voskoboynik O.Y., Berest G.G., Safronyuk S.L., Kovalenko S.I., Kamyshnyi O.M., Polishchuk N.M., Sinyak R.S., Katsev A.V. (2014). Synthesis and Antimicrobial Activity of 6-Thioxo-6,7-dihydro-2H-[1,2,4]triazino[2,3-c]-quinazolin-2-one Derivatives. Sci. Pharm..

[13] Froldi G. (2024). View on Metformin: Antidiabetic and Pleiotropic Effects, Pharmacokinetics, Side Effects, and Sex-Related Differences. Pharmaceuticals.

[14] Thakur S., Daley B., Klubo-Gwiezdzinska J. (2019). The role of an anti-diabetic drug metformin in the treatment of endocrine tumors. J. Mol. Endocrinol..

[15] Kamyshna I.I., Pavlovych L.B., Maslyanko V.A., Kamyshnyi A.M. (2021). Analysis of the transcriptional activity of genes of neuropeptides and their receptors in the blood of patients with thyroid pathology. J. Med. Life.

[16] Lyubomirskaya E.S., Kamyshnyi A.M., Krut Y.Y., Smiianov V.A., Fedoniuk L.Y., Romanyuk L.B., Kravets N.Y., Mochulska O.M. (2020). SNPs and transcriptional activity of genes of innate and adaptive immunity at the maternal-fetal interface in woman with preterm labour, associated with preterm premature rupture of membranes. Wiad. Lek..

[17] Kamyshna I.I., Pavlovych L.B., Sydorchuk L.P., Malyk I.V., Kamyshnyi A.M. (2021). BDNF blood serum linkage with BDNF gene polymorphism (rs6265) in thyroid pathology patients in the West-Ukrainian population. Endocr. Regul..

[18] Halabitska I., Babinets L. (2021). Different consequences of the treatment of osteoarthritis in gastrointestinal comorbidity with exocrine pancreatic insufficiency. Fam. Med. Prim. Care Rev..

[19] Baker C., Retzik-Stahr C., Singh V., Plomondon R., Anderson V., Rasouli N. (2021). Should metformin remain the first-line therapy for treatment of type 2 diabetes?. Ther. Adv. Endocrinol. Metab..

[20] Siavash Dastjerdi M., Tabbakhian M., Sabzghabaee A.M., Razavi N. (2017). Severity of Gastrointestinal Side Effects of Metformin Tablet Compared to Metformin Capsule in Type 2 Diabetes Mellitus Patients. J. Res. Pharm. Pract..

[21] Zemlyak O.S., Babinets L.S., Halabitska I.M. (2023). The Role of Endotoxicosis and Inflammation in Deepening the Pancreatic Functional Insufficiency in Chronic Pancreatitis in Combination with Type 2 Diabetes. Pol. Merkur. Lek. Organ Pol. Tow. Lek..

[22] Halabitska I., Oksenych V., Kamyshnyi O. (2024). Exploring the Efficacy of Alpha-Lipoic Acid in Comorbid Osteoarthritis and Type 2 Diabetes Mellitus. Nutrients.

[23] Lee H.S., Noh J.Y., Song J.Y., Cheong H.J., Kim W.J. (2023). Metformin reduces the risk of developing influenza A virus related cardiovascular disease. Heliyon.

[24] Ventura-López C., Cervantes-Luevano K., Aguirre-Sánchez J.S., Flores-Caballero J.C., Alvarez-Delgado C., Bernaldez-Sarabia J., Sánchez-Campos N., Lugo-Sánchez L.A., Rodríguez-Vázquez I.C., Sander-Padilla J.G. (2022). Treatment with metformin glycinate reduces SARS-CoV-2 viral load: An in vitro model and randomized, double-blind, Phase IIb clinical trial. Biomed. Pharmacother..

[25] Bhutta M.S., Gallo E.S., Borenstein R. (2021). Multifaceted Role of AMPK in Viral Infections. Cells.

[26] Parthasarathy H., Tandel D., Siddiqui A.H., Harshan K.H. (2023). Metformin suppresses SARS-CoV-2 in cell culture. Virus Res..

[27] Lin H., Ao H., Guo G., Liu M. (2023). The Role and Mechanism of Metformin in Inflammatory Diseases. J. Inflamm. Res..

[28] Dziedzic A., Saluk-Bijak J., Miller E., Bijak M. (2020). Metformin as a Potential Agent in the Treatment of Multiple Sclerosis. Int. J. Mol. Sci..

[29] Babinets L., Migenko B., Borovyk I., Halabitska I., Lobanets N., Onyskiv O. (2020). The role of cytocin imbalance in the development of man infertility. Wiad. Lek..

[30] Bharath L.P., Nikolajczyk B.S. (2021). The intersection of metformin and inflammation. Am. J. Physiol. Cell Physiol..

[31] Peairs A., Radjavi A., Davis S., Li L., Ahmed A., Giri S., Reilly C.M. (2009). Activation of AMPK inhibits inflammation in MRL/lpr mouse mesangial cells. Clin. Exp. Immunol..

[32] Amor S., Fernández Blanco L., Baker D. (2020). Innate immunity during SARS-CoV-2: Evasion strategies and activation trigger hypoxia and vascular damage. Clin. Exp. Immunol..

[33] Zasłona Z., O’Neill L.A.J. (2020). Cytokine-like Roles for Metabolites in Immunity. Mol. Cell.

[34] Marcucci F., Romeo E., Caserta C.A., Rumio C., Lefoulon F. (2020). Context-Dependent Pharmacological Effects of Metformin on the Immune System. Trends Pharmacol. Sci..

[35] Zong Y., Li H., Liao P., Chen L., Pan Y., Zheng Y., Zhang C., Liu D., Zheng M., Gao J. (2024). Mitochondrial dysfunction: Mechanisms and advances in therapy. Signal Transduct. Target. Ther..

[36] Benedetti F., Sorrenti V., Buriani A., Fortinguerra S., Scapagnini G., Zella D. (2020). Resveratrol, Rapamycin and Metformin as Modulators of Antiviral Pathways. Viruses.

[37] Plowman T.J., Christensen H., Aiges M., Fernandez E., Shah M.H., Ramana K.V. (2024). Anti-Inflammatory Potential of the Anti-Diabetic Drug Metformin in the Prevention of Inflammatory Complications and Infectious Diseases Including COVID-19: A Narrative Review. Int. J. Mol. Sci..

[38] Martin D.E., Cadar A.N., Bartley J.M. (2023). Old drug, new tricks: The utility of metformin in infection and vaccination responses to influenza and SARS-CoV-2 in older adults. Front. Aging.

[39] Khodadadi M., Jafari-Gharabaghlou D., Zarghami N. (2022). An update on mode of action of metformin in modulation of meta-inflammation and inflammaging. Pharmacol. Rep..

[40] Nojima I., Wada J. (2023). Metformin and Its Immune-Mediated Effects in Various Diseases. Int. J. Mol. Sci..

[41] Babinets L.S., Halabitska I.M., Kotsaba Y.Y., Borovyk I.O., Migenko B.O., Ryabokon S.S., Tsybulska L.S. (2018). The effect of the proteolisis’ system activity for the trophological status of patients with osteoarthrosis and excretory insufficiency of pancreas. Wiad. Lek..

[42] Suardi C., Cazzaniga E., Graci S., Dongo D., Palestini P. (2021). Link between Viral Infections, Immune System, Inflammation and Diet. Int. J. Environ. Res. Public Health.

[43] Cicchese J.M., Evans S., Hult C., Joslyn L.R., Wessler T., Millar J.A., Marino S., Cilfone N.A., Mattila J.T., Linderman J.J. (2018). Dynamic balance of pro- and anti-inflammatory signals controls disease and limits pathology. Immunol. Rev..

[44] Al-Qahtani A.A., Alhamlan F.S., Al-Qahtani A.A. (2024). Pro-Inflammatory and Anti-Inflammatory Interleukins in Infectious Diseases: A Comprehensive Review. Trop. Med. Infect. Dis..

[45] Sutter A., Landis D., Nugent K. (2024). Metformin has immunomodulatory effects which support its potential use as adjunctive therapy in tuberculosis. Indian J. Tuberc..

[46] Foretz M., Guigas B., Viollet B. (2023). Metformin: Update on mechanisms of action and repurposing potential. Nat. Rev. Endocrinol..

[47] Nagendra L., Bhattacharya S., Kalra S., Kapoor N. (2023). Metformin in COVID-19: Is There a Role Beyond Glycemic Control?. Int. J. Endocrinol. Metab..

[48] Drzewoski J., Hanefeld M. (2021). The Current and Potential Therapeutic Use of Metformin-The Good Old Drug. Pharmaceuticals.

[49] Mohammed I., Hollenberg M.D., Ding H., Triggle C.R. (2021). A Critical Review of the Evidence That Metformin Is a Putative Anti-Aging Drug That Enhances Healthspan and Extends Lifespan. Front. Endocrinol..

[50] Nogales A., Martínez-Sobrido L. (2016). Reverse Genetics Approaches for the Development of Influenza Vaccines. Int. J. Mol. Sci..

[51] Ashraf M.A., Raza M.A., Amjad M.N., Ud Din G., Yue L., Shen B., Chen L., Dong W., Xu H., Hu Y. (2024). A comprehensive review of influenza B virus, its biological and clinical aspects. Front. Microbiol..

[52] Yen F.S., Wei J.C., Shih Y.H., Hsu C.Y., Hsu C.C., Hwu C.M. (2022). Metformin Use before Influenza Vaccination May Lower the Risks of Influenza and Related Complications. Vaccines.

[53] Cummings T.H., Magagnoli J., Hardin J.W., Sutton S.S. (2022). Patients with Obesity and a History of Metformin Treatment Have Lower Influenza Mortality: A Retrospective Cohort Study. Pathogens.

[54] Greene E., Green C.L., Hurst J., MacIver N.J. (2024). Metformin use associated with lower rate of hospitalization for influenza in individuals with diabetes. Diabetes Obes. Metab..

[55] Thom R.E., D’Elia R.V. (2024). Future applications of host direct therapies for infectious disease treatment. Front. Immunol..

[56] Brandi P., Conejero L., Cueto F.J., Martínez-Cano S., Dunphy G., Gómez M.J., Relaño C., Saz-Leal P., Enamorado M., Quintas A. (2022). Trained immunity induction by the inactivated mucosal vaccine MV130 protects against experimental viral respiratory infections. Cell Rep..

[57] Goel S., Singh R., Singh V., Singh H., Kumari P., Chopra H., Sharma R., Nepovimova E., Valis M., Kuca K. (2022). Metformin: Activation of 5′ AMP-activated protein kinase and its emerging potential beyond anti-hyperglycemic action. Front. Genet..

[58] Martin D.E., Cadar A.N., Panier H., Torrance B.L., Kuchel G.A., Bartley J.M. (2023). The effect of metformin on influenza vaccine responses in nondiabetic older adults: A pilot trial. Immun. Ageing I A.

[59] Xia C., Wang T., Hahm B. (2024). Triggering Degradation of Host Cellular Proteins for Robust Propagation of Influenza Viruses. Int. J. Mol. Sci..

[60] Brown R.E., Chavez V., Hunter R.L. (2022). Morphoproteomic Features of Pulmonary Influenza A (H1N1) with Therapeutic Implications: A Case Study. Ann. Clin. Lab. Sci..

[61] Frasca D., Diaz A., Romero M., Blomberg B.B. (2021). Metformin Enhances B Cell Function and Antibody Responses of Elderly Individuals with Type-2 Diabetes Mellitus. Front. Aging.

[62] Hao W.R., Yang T.L., Lai Y.H., Lin K.J., Fang Y.A., Chen M.Y., Hsu M.H., Chiu C.C., Yang T.Y., Chen C.C. (2023). The Association between Influenza Vaccine and Risk of Chronic Kidney Disease/Dialysis in Patients with Hypertension. Vaccines.

[63] Saenwongsa W., Nithichanon A., Chittaganpitch M., Buayai K., Kewcharoenwong C., Thumrongwilainet B., Butta P., Palaga T., Takahashi Y., Ato M. (2020). Metformin-induced suppression of IFN-α via mTORC1 signalling following seasonal vaccination is associated with impaired antibody responses in type 2 diabetes. Sci. Rep..

[64] Yang A., Shi M., Wu H., Lau E.S.H., Ma R.C.W., Kong A.P.S., So W.Y., Luk A.O.Y., Chan J.C.N., Chow E. (2021). Long-term metformin use and risk of pneumonia and related death in type 2 diabetes: A registry-based cohort study. Diabetologia.

[65] Gedawy A., Al-Salami H., Dass C.R. (2020). Role of metformin in various pathologies: State-of-the-art microcapsules for improving its pharmacokinetics. Ther. Deliv..

[66] Kim J.W., Choe J.Y., Park S.H. (2022). Metformin and its therapeutic applications in autoimmune inflammatory rheumatic disease. Korean J. Intern. Med..

[67] Yang S., Wang L., Pan X., Liang Y., Zhang Y., Li J., Zhou B. (2022). 5-Methoxyflavone-induced AMPKα activation inhibits NF-κB and P38 MAPK signaling to attenuate influenza A virus-mediated inflammation and lung injury in vitro and in vivo. Cell. Mol. Biol. Lett..

[68] Hong S.W., Lee J., Park S.E., Rhee E.J., Park C.Y., Oh K.W., Park S.W., Lee W.Y. (2014). Activation of AMP-Activated Protein Kinase Attenuates Tumor Necrosis Factor-α-Induced Lipolysis via Protection of Perilipin in 3T3-L1 Adipocytes. Endocrinol. Metab..

[69] Uddin M.A., Akhter M.S., Kubra K.T., Siejka A., Barabutis N. (2021). Metformin in acute respiratory distress syndrome: An opinion. Exp. Gerontol..

[70] Fan S.Y., Zhao Z.C., Liu X.L., Peng Y.G., Zhu H.M., Yan S.F., Liu Y.J., Xie Q., Jiang Y., Zeng S.Z. (2024). Metformin Mitigates Sepsis-Induced Acute Lung Injury and Inflammation in Young Mice by Suppressing the S100A8/A9-NLRP3-IL-1β Signaling Pathway. J. Inflamm. Res..

[71] Khandia R., Dadar M., Munjal A., Dhama K., Karthik K., Tiwari R., Yatoo M.I., Iqbal H.M.N., Singh K.P., Joshi S.K. (2019). A Comprehensive Review of Autophagy and Its Various Roles in Infectious, Non-Infectious, and Lifestyle Diseases: Current Knowledge and Prospects for Disease Prevention, Novel Drug Design, and Therapy. Cells.

[72] Tao S., Drexler I. (2020). Targeting Autophagy in Innate Immune Cells: Angel or Demon During Infection and Vaccination?. Front. Immunol..

[73] Pehote G., Vij N. (2020). Autophagy Augmentation to Alleviate Immune Response Dysfunction, and Resolve Respiratory and COVID-19 Exacerbations. Cells.

[74] Chen T., Tu S., Ding L., Jin M., Chen H., Zhou H. (2023). The role of autophagy in viral infections. J. Biomed. Sci..

[75] Abdelaziz D.H., Thapa S., Abdulrahman B., Vankuppeveld L., Schatzl H.M. (2020). Metformin reduces prion infection in neuronal cells by enhancing autophagy. Biochem. Biophys. Res. Commun..

[76] Zhang S., Zou W., Leng Y., Mu Z., Zhan L. (2024). Neuroprotective Effects of Metformin on Cerebral Ischemia-Reperfusion Injury: Modulation of JNK and p38 MAP Kinase Signaling Pathways. Cell Biochem. Biophys..

[77] Rothenburg S., Brennan G. (2020). Species-Specific Host-Virus Interactions: Implications for Viral Host Range and Virulence. Trends Microbiol..

[78] Weitzman M.D., Fradet-Turcotte A. (2018). Virus DNA Replication and the Host DNA Damage Response. Annu. Rev. Virol..

[79] Shaikh S.R., MacIver N.J., Beck M.A. (2022). Obesity Dysregulates the Immune Response to Influenza Infection and Vaccination Through Metabolic and Inflammatory Mechanisms. Annu. Rev. Nutr..

[80] Hulme K.D., Noye E.C., Short K.R., Labzin L.I. (2021). Dysregulated Inflammation During Obesity: Driving Disease Severity in Influenza Virus and SARS-CoV-2 Infections. Front. Immunol..

[81] Chen X., Guo H., Qiu L., Zhang C., Deng Q., Leng Q. (2020). Immunomodulatory and Antiviral Activity of Metformin and Its Potential Implications in Treating Coronavirus Disease 2019 and Lung Injury. Front. Immunol..

[82] Moreira D., Silvestre R., Cordeiro-da-Silva A., Estaquier J., Foretz M., Viollet B. (2016). AMP-activated Protein Kinase As a Target For Pathogens: Friends Or Foes?. Curr. Drug Targets.

[83] Prantner D., Perkins D.J., Vogel S.N. (2017). AMP-activated Kinase (AMPK) Promotes Innate Immunity and Antiviral Defense through Modulation of Stimulator of Interferon Genes (STING) Signaling. J. Biol. Chem..

[84] Ren D., Qin G., Zhao J., Sun Y., Zhang B., Li D., Wang B., Jin X., Wu H. (2020). Metformin activates the STING/IRF3/IFN-β pathway by inhibiting AKT phosphorylation in pancreatic cancer. Am. J. Cancer Res..

[85] Pereira G., Leão A., Erustes A.G., Morais I.B.M., Vrechi T.A.M., Zamarioli L.D.S., Pereira C.A.S., Marchioro L.O., Sperandio L.P., Lins I.V.F. (2021). Pharmacological Modulators of Autophagy as a Potential Strategy for the Treatment of COVID-19. Int. J. Mol. Sci..

[86] Checa J., Aran J.M. (2020). Reactive Oxygen Species: Drivers of Physiological and Pathological Processes. J. Inflamm. Res..

[87] Li M., Zhao Z., Yi J. (2024). Biomaterials Designed to Modulate Reactive Oxygen Species for Enhanced Bone Regeneration in Diabetic Conditions. J. Funct. Biomater..

[88] Salvatore T., Pafundi P.C., Galiero R., Rinaldi L., Caturano A., Vetrano E., Aprea C., Albanese G., Di Martino A., Ricozzi C. (2020). Can Metformin Exert as an Active Drug on Endothelial Dysfunction in Diabetic Subjects?. Biomedicines.

[89] Luo P., Qiu L., Liu Y., Liu X.L., Zheng J.L., Xue H.Y., Liu W.H., Liu D., Li J. (2020). Metformin Treatment Was Associated with Decreased Mortality in COVID-19 Patients with Diabetes in a Retrospective Analysis. Am. J. Trop. Med. Hyg..

[90] Torunoglu S.T., Zajda A., Tampio J., Markowicz-Piasecka M., Huttunen K.M. (2023). Metformin derivatives—Researchers’ friends or foes?. Biochem. Pharmacol..

[91] Bartee E., McFadden G. (2013). Cytokine synergy: An underappreciated contributor to innate anti-viral immunity. Cytokine.

[92] Halabitska I., Babinets L., Kotsaba Y. (2021). PATHOGENETIC FEATURES OF COMORBIDITY OF PRIMARY OSTEOARTHRITIS AND DISEASES WITH EXOCRINE PANCREATIC INSUFFICIENCY. Georgian Med. News.

[93] Li J.H., Hsin P.Y., Hsiao Y.C., Chen B.J., Zhuang Z.Y., Lee C.W., Lee W.J., Vo T.T.T., Tseng C.F., Tseng S.F. (2024). A Narrative Review: Repurposing Metformin as a Potential Therapeutic Agent for Oral Cancer. Cancers.

[94] Fert A., Richard J., Raymond Marchand L., Planas D., Routy J.P., Chomont N., Finzi A., Ancuta P. (2024). Metformin facilitates viral reservoir reactivation and their recognition by anti-HIV-1 envelope antibodies. iScience.

[95] Szewczuk M., Boguszewska K., Kaźmierczak-Barańska J., Karwowski B.T. (2020). The role of AMPK in metabolism and its influence on DNA damage repair. Mol. Biol. Rep..

[96] de Marañón A.M., Díaz-Pozo P., Canet F., Díaz-Morales N., Abad-Jiménez Z., López-Domènech S., Vezza T., Apostolova N., Morillas C., Rocha M. (2022). Metformin modulates mitochondrial function and mitophagy in peripheral blood mononuclear cells from type 2 diabetic patients. Redox Biol..

[97] Jing W., Liu C., Su C., Liu L., Chen P., Li X., Zhang X., Yuan B., Wang H., Du X. (2023). Role of reactive oxygen species and mitochondrial damage in rheumatoid arthritis and targeted drugs. Front. Immunol..

[98] Kozlov A.V., Javadov S., Sommer N. (2024). Cellular ROS and Antioxidants: Physiological and Pathological Role. Antioxidants.

[99] Chow E., Yang A., Chung C.H.L., Chan J.C.N. (2022). A Clinical Perspective of the Multifaceted Mechanism of Metformin in Diabetes, Infections, Cognitive Dysfunction, and Cancer. Pharmaceuticals.

[100] Justice J.N., Gubbi S., Kulkarni A.S., Bartley J.M., Kuchel G.A., Barzilai N. (2021). A geroscience perspective on immune resilience and infectious diseases: A potential case for metformin. GeroScience.

[101] Varghese E., Samuel S.M., Liskova A., Kubatka P., Büsselberg D. (2021). Diabetes and coronavirus (SARS-CoV-2): Molecular mechanism of Metformin intervention and the scientific basis of drug repurposing. PLoS Pathog..

[102] Samuel S.M., Varghese E., Büsselberg D. (2021). Therapeutic Potential of Metformin in COVID-19: Reasoning for Its Protective Role. Trends Microbiol..

[103] Buchynskyi M., Oksenych V., Kamyshna I., Vorobets I., Halabitska I., Kamyshnyi O. (2024). Modulatory Roles of AHR, FFAR2, FXR, and TGR5 Gene Expression in Metabolic-Associated Fatty Liver Disease and COVID-19 Outcomes. Viruses.

[104] Buchynskyi M., Oksenych V., Kamyshna I., Budarna O., Halabitska I., Petakh P., Kamyshnyi O. (2024). Genomic insight into COVID-19 severity in MAFLD patients: A single-center prospective cohort study. Front. Genet..

[105] Buchynskyi M., Oksenych V., Kamyshna I., Vari S.G., Kamyshnyi A. (2023). Genetic Predictors of Comorbid Course of COVID-19 and MAFLD: A Comprehensive Analysis. Viruses.

[106] Pedrosa A.R., Martins D.C., Rizzo M., Silva-Nunes J. (2023). Metformin in SARS-CoV-2 infection: A hidden path—From altered inflammation to reduced mortality. A review from the literature. J. Diabetes Its Complicat..

[107] Bramante C.T., Beckman K.B., Mehta T., Karger A.B., Odde D.J., Tignanelli C.J., Buse J.B., Johnson D.M., Watson R.H.B., Daniel J.J. (2023). Metformin reduces SARS-CoV-2 in a Phase 3 Randomized Placebo Controlled Clinical Trial. medRxiv Prepr. Serv. Health Sci..

[108] Zhao M. (2020). Cytokine storm and immunomodulatory therapy in COVID-19: Role of chloroquine and anti-IL-6 monoclonal antibodies. Int. J. Antimicrob. Agents.

[109] Zhang W., Qin C., Fei Y., Shen M., Zhou Y., Zhang Y., Zeng X., Zhang S. (2022). Anti-inflammatory and immune therapy in severe coronavirus disease 2019 (COVID-19) patients: An update. Clin. Immunol..

[110] Halabitska I., Petakh P., Oksenych V., Kamyshnyi O. (2024). Predictive analysis of osteoarthritis and chronic pancreatitis comorbidity: Complications and risk factors. Front. Endocrinol..

[111] Rena G., Hardie D.G., Pearson E.R. (2017). The mechanisms of action of metformin. Diabetologia.

[112] Putilin D.A., Evchenko S.Y., Fedoniuk L.Y., Tokarskyy O.S., Kamyshny O.M., Migenko L.M., Andreychyn S.M., Hanberher I.I., Bezruk T.O. (2020). The Influence of Metformin to the Transcriptional Activity of the mTOR and FOX3 Genes in Parapancreatic Adipose Tissue of Streptozotocin-Induced Diabetic Rats. J. Med. Life.

[113] Bramante C.T., Buse J.B., Liebovitz D.M., Nicklas J.M., Puskarich M.A., Cohen K., Belani H.K., Anderson B.J., Huling J.D., Tignanelli C.J. (2023). Outpatient treatment of COVID-19 and incidence of post-COVID-19 condition over 10 months (COVID-OUT): A multicentre, randomised, quadruple-blind, parallel-group, phase 3 trial. Lancet Infect. Dis..

[114] Kamyshnyi O., Matskevych V., Lenchuk T., Strilbytska O., Storey K., Lushchak O. (2021). Metformin to decrease COVID-19 severity and mortality: Molecular mechanisms and therapeutic potential. Biomed. Pharmacother..

[115] Malhotra A., Hepokoski M., McCowen K.C., Shyy J.Y.J. (2020). ACE2, Metformin, and COVID-19. iScience.

[116] Bramante C.T., Huling J.D., Tignanelli C.J., Buse J.B., Liebovitz D.M., Nicklas J.M., Cohen K., Puskarich M.A., Belani H.K., Proper J.L. (2022). Randomized Trial of Metformin, Ivermectin, and Fluvoxamine for COVID-19. N. Engl. J. Med..

[117] Petakh P., Kamyshna I., Oksenych V., Kainov D., Kamyshnyi A. (2023). Metformin Therapy Changes Gut Microbiota Alpha-Diversity in COVID-19 Patients with Type 2 Diabetes: The Role of SARS-CoV-2 Variants and Antibiotic Treatment. Pharmaceuticals.

[118] Lalau J.D., Al-Salameh A., Hadjadj S., Goronflot T., Wiernsperger N., Pichelin M., Allix I., Amadou C., Bourron O., Duriez T. (2021). Metformin use is associated with a reduced risk of mortality in patients with diabetes hospitalised for COVID-19. Diabetes Metab..

[119] Petakh P., Kamyshna I., Kamyshnyi A. (2024). Gene expression of protein kinase AMP-activated catalytic subunit alpha 1 (PRKAA1), solute carrier family 2 member 1 (SLC2A1) and mechanistic target of rapamycin (MTOR) in metformin-treated type 2 diabetes patients with COVID-19: Impact on inflammation markers. Inflammopharmacology.

[120] Bramante C.T., Beckman K.B., Mehta T., Karger A.B., Odde D.J., Tignanelli C.J., Buse J.B., Johnson D.M., Watson R.H.B., Daniel J.J. (2024). Favorable Antiviral Effect of Metformin on SARS-CoV-2 Viral Load in a Randomized, Placebo-Controlled Clinical Trial of COVID-19. Clin. Infect. Dis. Off. Publ. Infect. Dis. Soc. Am..

[121] Boulware D.R., Murray T.A., Proper J.L., Tignanelli C.J., Buse J.B., Liebovitz D.M., Nicklas J.M., Cohen K., Puskarich M.A., Belani H.K. (2023). Impact of Severe Acute Respiratory Syndrome Coronavirus 2 (SARS-CoV-2) Vaccination and Booster on Coronavirus Disease 2019 (COVID-19) Symptom Severity Over Time in the COVID-OUT Trial. Clin. Infect. Dis. Off. Publ. Infect. Dis. Soc. Am..

[122] Petakh P., Griga V., Mohammed I.B., Loshak K., Poliak I., Kamyshnyiy A. (2022). Effects of Metformin, Insulin on Hematological Parameters of COVID-19 Patients with Type 2 Diabetes. Med. Arch..

[123] Petrelli F., Grappasonni I., Nguyen C.T.T., Tesauro M., Pantanetti P., Xhafa S., Cangelosi G. (2023). Metformin and COVID-19: A systematic review of systematic reviews with meta-analysis. Acta Bio-Medica Atenei Parm..

[124] Silverii G.A., Fumagalli C., Rozzini R., Milani M., Mannucci E., Marchionni N. (2024). Is Metformin Use Associated with a More Favorable COVID-19 Course in People with Diabetes?. J. Clin. Med..

[125] Petakh P., Oksenych V., Kamyshnyi A. (2023). The F/B ratio as a biomarker for inflammation in COVID-19 and T2D: Impact of metformin. Biomed. Pharmacother..

[126] Miguel V., Rey-Serra C., Tituaña J., Sirera B., Alcalde-Estévez E., Herrero J.I., Ranz I., Fernández L., Castillo C., Sevilla L. (2023). Enhanced fatty acid oxidation through metformin and baicalin as therapy for COVID-19 and associated inflammatory states in lung and kidney. Redox Biol..

[127] Petakh P., Kobyliak N., Kamyshnyi A. (2023). Gut microbiota in patients with COVID-19 and type 2 diabetes: A culture-based method. Front. Cell. Infect. Microbiol..

[128] Hou Y., Yang Z., Xiang B., Liu J., Geng L., Xu D., Zhan M., Xu Y., Zhang B. (2024). Metformin is a potential therapeutic for COVID-19/LUAD by regulating glucose metabolism. Sci. Rep..

[129] Al-Kuraishy H.M., Al-Gareeb A.I., El Kholy A.A., El-Khateeb E., Alexiou A., Papadakis M., Elekhnawy E., Alsubaie N., Hamad R.S., Batiha G.E. (2023). The potential therapeutic effect of metformin in type 2 diabetic patients with severe COVID-19. Eur. Rev. Med. Pharmacol. Sci..

[130] Petakh P., Kamyshna I., Oksenych V., Kamyshnyi O. (2024). Metformin Alters mRNA Expression of FOXP3, RORC, and TBX21 and Modulates Gut Microbiota in COVID-19 Patients with Type 2 Diabetes. Viruses.

[131] Somasundaram M., Mathew S.K., Paul S., Kurian S.J., Kunhikatta V., Karanth S., Shetty S., Kudru C.U., Manu M.K., Saravu K. (2024). Metformin use and its association with various outcomes in COVID-19 patients with diabetes mellitus: A retrospective cohort study in a tertiary care facility. Ann. Med..

[132] Qiu S., Hubbard A.E., Gutiérrez J.P., Pimpale G., Juárez-Flores A., Ghosh R., de Jesús Ascencio-Montiel I., Bertozzi S.M. (2024). Estimating the effect of realistic improvements of metformin adherence on COVID-19 mortality using targeted machine learning. Glob. Epidemiol..

[133] Lockwood T.D. (2024). Coordination chemistry suggests that independently observed benefits of metformin and Zn^2+^ against COVID-19 are not independent. Biometals Int. J. Role Met. Ions Biol. Biochem. Med..

[134] Harmon D.C., Levene J.A., Rutlen C.L., White E.S., Freeman I.R., Lapidus J.A. (2024). Preadmission Metformin Use Is Associated with Reduced Mortality in Patients with Diabetes Mellitus Hospitalized with COVID-19. J. Gen. Intern. Med..

[135] Lewandowski Ł., Bronowicka-Szydełko A., Rabczyński M., Bednarska-Chabowska D., Adamiec-Mroczek J., Doroszko A., Trocha M., Kujawa K., Matera-Witkiewicz A., Kuźnik E. (2024). Insulin and Metformin Administration: Unravelling the Multifaceted Association with Mortality across Various Clinical Settings Considering Type 2 Diabetes Mellitus and COVID-19. Biomedicines.

[136] De Jesús-González L.A., Del Ángel R.M., Palacios-Rápalo S.N., Cordero-Rivera C.D., Rodríguez-Carlos A., Trujillo-Paez J.V., Farfan-Morales C.N., Osuna-Ramos J.F., Reyes-Ruiz J.M., Rivas-Santiago B. (2024). A Dual Pharmacological Strategy against COVID-19: The Therapeutic Potential of Metformin and Atorvastatin. Microorganisms.

[137] Wiernsperger N., Al-Salameh A., Cariou B., Lalau J.D. (2022). Protection by metformin against severe COVID-19: An in-depth mechanistic analysis. Diabetes Metab..

[138] To E.E., Erlich J.R., Liong F., Luong R., Liong S., Esaq F., Oseghale O., Anthony D., McQualter J., Bozinovski S. (2020). Mitochondrial Reactive Oxygen Species Contribute to Pathological Inflammation During Influenza A Virus Infection in Mice. Antioxid. Redox Signal..

[139] Petakh P., Isevych V., Mohammed I., Loshak K., Poliak I., Kamyshnyi O. (2022). Association between Use of Metformin and Insulin with Hematological Parameters in COVID-19 Patients with Type 2 Diabetes: A Single Center, Cross-Sectional Study. Clin. Diabetol..

[140] Deaton A., Cartwright N. (2018). Understanding and misunderstanding randomized controlled trials. Soc. Sci. Med..

[141] Wihandani D.M., Purwanta M.L.A., Mulyani W.R.W., Putra I., Supadmanaba I.G.P. (2023). New-onset diabetes in COVID-19: The molecular pathogenesis. BioMedicine.

[142] Xie L., Zhang Z., Wang Q., Chen Y., Lu D., Wu W. (2021). COVID-19 and Diabetes: A Comprehensive Review of Angiotensin Converting Enzyme 2, Mutual Effects and Pharmacotherapy. Front. Endocrinol..

[143] Buchynskyi M., Kamyshna I., Oksenych V., Zavidniuk N., Kamyshnyi A. (2023). The Intersection of COVID-19 and Metabolic-Associated Fatty Liver Disease: An Overview of the Current Evidence. Viruses.

[144] Nafisa A., Gray S.G., Cao Y., Wang T., Xu S., Wattoo F.H., Barras M., Cohen N., Kamato D., Little P.J. (2018). Endothelial function and dysfunction: Impact of metformin. Pharmacol. Ther..

[145] Sydorchuk L., Dzhuryak V., Sydorchuk A., Levytska S., Petrynych V., Knut R., Kshanovska A., Iftoda O., Tkachuk O., Kyfiak P. (2020). The cytochrome 11B2 aldosterone synthase gene rs1799998 single nucleotide polymorphism determines elevated aldosterone, higher blood pressure, and reduced glomerular filtration, especially in diabetic female patients. Endocr. Regul..

[146] Repchuk Y., Sydorchuk L.P., Sydorchuk A.R., Fedonyuk L.Y., Kamyshnyi O., Korovenkova O., Plehutsa I.M., Dzhuryak V.S., Myshkovskii Y.M., Iftoda O.M. (2021). Linkage of blood pressure, obesity and diabetes mellitus with angiotensinogen gene (AGT 704T>C/rs699) polymorphism in hypertensive patients. Bratisl. Lek. Listy.

[147] Scheen A.J. (2020). Metformin and COVID-19: From cellular mechanisms to reduced mortality. Diabetes Metab..

[148] Petakh P., Kamyshna I., Nykyforuk A., Yao R., Imbery J.F., Oksenych V., Korda M., Kamyshnyi A. (2022). Immunoregulatory Intestinal Microbiota and COVID-19 in Patients with Type Two Diabetes: A Double-Edged Sword. Viruses.

[149] Asar T., Al-Abbasi F., Sheikh R., Zeyadi M., Nadeem M., Naqvi S., Kumar V., Anwar F. (2024). Metformin’s dual impact on Gut microbiota and cardiovascular health: A comprehensive analysis. Biomed. Pharmacother..

[150] Petakh P., Kamyshna I., Kamyshnyi A. (2023). Unveiling the potential pleiotropic effects of metformin in treating COVID-19: A comprehensive review. Front. Mol. Biosci..

[151] Buchynskyi M., Kamyshna I., Lyubomirskaya K., Moshynets O., Kobyliak N., Oksenych V., Kamyshnyi A. (2023). Efficacy of interferon alpha for the treatment of hospitalized patients with COVID-19: A meta-analysis. Front. Immunol..

[152] Kamyshnyi A., Koval H., Kobevko O., Buchynskyi M., Oksenych V., Kainov D., Lyubomirskaya K., Kamyshna I., Potters G., Moshynets O. (2023). Therapeutic Effectiveness of Interferon-α2b against COVID-19 with Community-Acquired Pneumonia: The Ukrainian Experience. Int. J. Mol. Sci..

[153] Buchynskyi M., Oksenych V., Kamyshna I., Kamyshnyi O. (2024). Exploring Paxlovid Efficacy in COVID-19 Patients with MAFLD: Insights from a Single-Center Prospective Cohort Study. Viruses.

[154] Ibrahim S., Lowe J.R., Bramante C.T., Shah S., Klatt N.R., Sherwood N., Aronne L., Puskarich M., Tamariz L., Palacio A. (2021). Metformin and COVID-19: Focused Review of Mechanisms and Current Literature Suggesting Benefit. Front. Endocrinol..

[155] Zangiabadian M., Nejadghaderi S.A., Zahmatkesh M.M., Hajikhani B., Mirsaeidi M., Nasiri M.J. (2021). The Efficacy and Potential Mechanisms of Metformin in the Treatment of COVID-19 in the Diabetics: A Systematic Review. Front. Endocrinol..

[156] Ganesh A., Randall M.D. (2022). Does metformin affect outcomes in COVID-19 patients with new or pre-existing diabetes mellitus? A systematic review and meta-analysis. Br. J. Clin. Pharmacol..

[157] Sardu C., Marfella R., Prattichizzo F., La Grotta R., Paolisso G., Ceriello A. (2022). Effect of Hyperglycemia on COVID-19 Outcomes: Vaccination Efficacy, Disease Severity, and Molecular Mechanisms. J. Clin. Med..

[158] Zaongo S.D., Chen Y. (2023). Metformin may be a viable adjunctive therapeutic option to potentially enhance immune reconstitution in HIV-positive immunological non-responders. Chin. Med. J..

[159] Babinets L., Halabitska I. (2020). Chronic inflammatory process and bone tissue changes in patients with osteoarthritis and exocrine pancreatic insufficiency. Lek. Obz..

[160] Chew G.M., Padua A.J.P., Chow D.C., Souza S.A., Clements D.M., Corley M.J., Pang A.P.S., Alejandria M.M., Gerschenson M., Shikuma C.M. (2021). Effects of Brief Adjunctive Metformin Therapy in Virologically Suppressed HIV-Infected Adults on Polyfunctional HIV-Specific CD8 T Cell Responses to PD-L1 Blockade. AIDS Res. Hum. Retroviruses.

[161] Pollak M. (2017). The effects of metformin on gut microbiota and the immune system as research frontiers. Diabetologia.

[162] Hasanvand A. (2022). The role of AMPK-dependent pathways in cellular and molecular mechanisms of metformin: A new perspective for treatment and prevention of diseases. Inflammopharmacology.

[163] Choi Y.K., Park K.G. (2013). Metabolic roles of AMPK and metformin in cancer cells. Mol. Cells.

[164] Kifle Z.D., Woldeyohanis A.E., Demeke C.A. (2021). A review on protective roles and potential mechanisms of metformin in diabetic patients diagnosed with COVID-19. Metab. Open.

[165] Schuiveling M., Vazirpanah N., Radstake T., Zimmermann M., Broen J. (2018). Metformin, A New Era for an Old Drug in the Treatment of Immune Mediated Disease?. Curr. Drug Targets.

[166] McCabe L., Burns J.E., Latifoltojar A., Post F.A., Fox J., Pool E., Waters A., Santana B., Garvey L., Johnson M. (2024). MAVMET trial: Maraviroc and/or metformin for metabolic dysfunction associated fatty liver disease in adults with suppressed HIV. AIDS.

[167] Rezaei S., Timani K., He J. (2023). Metformin Treatment Leads to Increased HIV Transcription and Gene Expression through Increased CREB Phosphorylation and Recruitment to the HIV LTR Promoter. Aging Dis..

[168] McCrea J.B., Patel M., Liu Y., Vargo R., Witter R., Litovsky A., Stoch S.A., Iwamoto M., Matthews R.P. (2024). Pharmacokinetics of Atorvastatin and Metformin after Coadministration with Islatravir in Healthy Adults. J. Clin. Pharmacol..

[169] Corley M.J., Pang A.P.S., Shikuma C.M., Ndhlovu L.C. (2024). Cell-type specific impact of metformin on monocyte epigenetic age reversal in virally suppressed older people living with HIV. Aging Cell.

[170] Nguyen D., Miao X., Taskar K., Magee M., Gorycki P., Moore K., Tai G. (2024). No dose adjustment of metformin or substrates of organic cation transporters (OCT)1 and OCT2 and multidrug and toxin extrusion protein (MATE)1/2K with fostemsavir coadministration based on modeling approaches. Pharmacol. Res. Perspect..

[171] Mhlanga N.L., Netangaheni T.R. (2024). Interventions for Type 2 Diabetes reduction among older people living with HIV in Harare. S. Afr. Fam. Pract..

[172] Hurbans N., Naidoo P. (2024). Comorbidity and concomitant medication use in an integrase strand transfer inhibitor naïve cohort on first-line dolutegravir-based antiretroviral therapy. Pan Afr. Med. J..

[173] Duan W., Ding Y., Yu X., Ma D., Yang B., Li Y., Huang L., Chen Z., Zheng J., Yang C. (2019). Metformin mitigates autoimmune insulitis by inhibiting Th1 and Th17 responses while promoting Treg production. Am. J. Transl. Res..

[174] Smigiel K., Srivastava S., Stolley J., Campbell D. (2014). Regulatory T-cell homeostasis: Steady-state maintenance and modulation during inflammation. Immunol. Rev..

[175] Jenabian M.A., Ancuta P., Gilmore N., Routy J.P. (2012). Regulatory T cells in HIV infection: Can immunotherapy regulate the regulator?. Clin. Dev. Immunol..

[176] Esmail Nia G., Mohammadi M., Sharifizadeh M., Ghalamfarsa G., Bolhassani A. (2024). The role of T regulatory cells in the immunopathogenesis of HIV: Clinical implications. Braz. J. Infect. Dis..

[177] Bai B., Chen H. (2021). Metformin: A Novel Weapon Against Inflammation. Front. Pharmacol..

[178] Wang J., Zhu L., Hu K., Tang Y., Zeng X., Liu J., Xu J. (2017). Effects of metformin treatment on serum levels of C-reactive protein and interleukin-6 in women with polycystic ovary syndrome: A meta-analysis: A PRISMA-compliant article. Medicine.

[179] Obare L.M., Temu T., Mallal S.A., Wanjalla C.N. (2024). Inflammation in HIV and Its Impact on Atherosclerotic Cardiovascular Disease. Circ. Res..

[180] Lv T., Cao W., Li T. (2021). HIV-Related Immune Activation and Inflammation: Current Understanding and Strategies. J. Immunol. Res..

[181] Ouyang J., Isnard S., Lin J., Fombuena B., Marette A., Routy B., Chen Y., Routy J.P. (2020). Metformin effect on gut microbiota: Insights for HIV-related inflammation. AIDS Res. Ther..

[182] So-Armah K., Benjamin L.A., Bloomfield G.S., Feinstein M.J., Hsue P., Njuguna B., Freiberg M.S. (2020). HIV and cardiovascular disease. Lancet HIV.

[183] Topol I., Kamyshny A. (2013). Study of expression of TLR2, TLR4 and transckription factor NF-kB structures of galt of rats in the conditions of the chronic social stress and modulation of structure of intestinal microflora. Georgian Med. News.

[184] MacCann R., Landay A.L., Mallon P.W.G. (2023). HIV and comorbidities—The importance of gut inflammation and the kynurenine pathway. Curr. Opin. HIV AIDS.

[185] Ponte R., Mehraj V., Ghali P., Couëdel-Courteille A., Cheynier R., Routy J.P. (2016). Reversing Gut Damage in HIV Infection: Using Non-Human Primate Models to Instruct Clinical Research. EBioMedicine.

[186] Fakharian F., Thirugnanam S., Welsh D.A., Kim W.K., Rappaport J., Bittinger K., Rout N. (2023). The Role of Gut Dysbiosis in the Loss of Intestinal Immune Cell Functions and Viral Pathogenesis. Microorganisms.

[187] Topol I.A., Kamyshny A.M., Abramov A.V., Kolesnik Y.M. (2014). Expression of XBP1 in lymphocytes of the small intestine in rats under chronic social stress and modulation of intestinal microflora composition. Fiziolohichnyi Zhurnal.

[188] Mazzuti L., Turriziani O., Mezzaroma I. (2023). The Many Faces of Immune Activation in HIV-1 Infection: A Multifactorial Interconnection. Biomedicines.

[189] Mu W., Patankar V., Kitchen S., Zhen A. (2024). Examining Chronic Inflammation, Immune Metabolism, and T Cell Dysfunction in HIV Infection. Viruses.

[190] Nasri H., Rafieian-Kopaei M. (2014). Metformin: Current knowledge. J. Res. Med. Sci..

[191] Routy J.-P., Isnard S., Mehraj V., Ostrowski M., Chomont N., Ancuta P., Ponte R., Planas D., Dupuy F., Angel J. (2019). Effect of metformin on the size of the HIV reservoir in non-diabetic ART-treated individuals: Single-arm non-randomised Lilac pilot study protocol. BMJ Open.

[192] Nelson A.G., Zhang X., Ganapathi U., Szekely Z., Flexner C.W., Owen A., Sinko P.J. (2015). Drug delivery strategies and systems for HIV/AIDS pre-exposure prophylaxis and treatment. J. Control. Release.

[193] Nicol M.R., Adams J.L., Kashuba A.D. (2013). HIV PrEP Trials: The Road to Success. Clin. Investig..

[194] Deeks S.G., Archin N., Cannon P., Collins S., Jones R.B., de Jong M., Lambotte O., Lamplough R., Ndung’u T., Sugarman J. (2021). Research priorities for an HIV cure: International AIDS Society Global Scientific Strategy 2021. Nat. Med..

[195] Kristófi R., Eriksson J. (2021). Metformin as an anti-inflammatory agent: A short review. J. Endocrinol..

[196] Halabitska I., Babinets L., Oksenych V., Kamyshnyi O. (2024). Diabetes and Osteoarthritis: Exploring the Interactions and Therapeutic Implications of Insulin, Metformin, and GLP-1-Based Interventions. Biomedicines.

[197] Kanda T., Yokosuka O., Omata M. (2013). Hepatitis C virus and hepatocellular carcinoma. Biology.

[198] Coppola N., Vatiero L.M., Sagnelli E. (2005). HCV genotype 2 as a risk factor for reactivation of chronic HCV infection. Gut.

[199] Babinets L.S., Shaihen O.R., Homyn H.O., Halabitska I.M. (2019). Specific aspects of clinical course in case of combination of chronic pancreatitis and concomitant viral hepatitis C. Wiad. Lek..

[200] Papadakos S.P., Ferraro D., Carbone G., Frampton A.E., Vennarecci G., Kykalos S., Schizas D., Theocharis S., Machairas N. (2023). The Emerging Role of Metformin in the Treatment of Hepatocellular Carcinoma: Is There Any Value in Repurposing Metformin for HCC Immunotherapy?. Cancers.

[201] Kwo P.Y. (2024). Metformin and statins and their role in reducing hepatocellular carcinoma risk: Randomized trials are needed: Editorial on “Metformin and statins reduce hepatocellular carcinoma risk in chronic hepatitis C patients with failed antiviral therapy”. Clin. Mol. Hepatol..

[202] Landis D., Sutter A., Khemka S., Songtanin B., Nichols J., Nugent K. (2024). Metformin as adjuvant treatment in hepatitis C virus infections and associated complications. Am. J. Med. Sci..

[203] Leslie J., Geh D., Elsharkawy A.M., Mann D.A., Vacca M. (2022). Metabolic dysfunction and cancer in HCV: Shared pathways and mutual interactions. J. Hepatol..

[204] Stephenne X., Foretz M., Taleux N., van der Zon G.C., Sokal E., Hue L., Viollet B., Guigas B. (2011). Metformin activates AMP-activated protein kinase in primary human hepatocytes by decreasing cellular energy status. Diabetologia.

[205] Tsai P.C., Kuo H.T., Hung C.H., Tseng K.C., Lai H.C., Peng C.Y., Wang J.H., Chen J.J., Lee P.L., Chien R.N. (2023). Metformin reduces hepatocellular carcinoma incidence after successful antiviral therapy in patients with diabetes and chronic hepatitis C in Taiwan. J. Hepatol..

[206] Shimada S., Kamiyama T., Orimo T., Nagatsu A., Kamachi H., Taketomi A. (2021). High HbA1c is a risk factor for complications after hepatectomy and influences for hepatocellular carcinoma without HBV and HCV infection. Hepatobiliary Surg. Nutr..

[207] Lin D., Reddy V., Osman H., Lopez A., Koksal A.R., Rhadhi S.M., Dash S., Aydin Y. (2021). Additional Inhibition of Wnt/β-Catenin Signaling by Metformin in DAA Treatments as a Novel Therapeutic Strategy for HCV-Infected Patients. Cells.

[208] Berk J., Lorigiano T.J., Sulkowski M., Mixter S. (2020). Replacing Insulin with Anti-Virals: A Clinical Vignette on Diabetes and HCV Treatment. AACE Clin. Case Rep..

[209] Abdel Monem M.S., Farid S.F., Abbassi M.M., Youssry I., Andraues N.G., Hassany M., Selim Y.M.M., El-Sayed M.H. (2021). The potential hepatoprotective effect of metformin in hepatitis C virus-infected adolescent patients with beta thalassemia major: Randomised clinical trial. Int. J. Clin. Pract..

[210] Valenti L., Pelusi S., Aghemo A., Gritti S., Pasulo L., Bianco C., Iegri C., Cologni G., Degasperi E., D’Ambrosio R. (2022). Dysmetabolism, Diabetes and Clinical Outcomes in Patients Cured of Chronic Hepatitis C: A Real-Life Cohort Study. Hepatol. Commun..

[211] Rodríguez-Escaja C., Navascues C.Á., González-Diéguez L., Cadahía V., Varela M., de Jorge M., Castaño-García A., Rodríguez M. (2021). Diabetes is not associated with an increased risk of hepatocellular carcinoma in patients with alcoholic or hepatitis C virus cirrhosis. Rev. Esp. De Enfermedades Dig..

[212] Thomaz M.L., Vieira C.P., Caris J.A., Marques M.P., Rocha A., Paz T.A., Rezende R.E.F., Lanchote V.L. (2024). Liver Fibrosis Stages Affect Organic Cation Transporter 1/2 Activities in Hepatitis C Virus-Infected Patients. Pharmaceuticals.

[213] Chung W., Wong K., Ravindranayagam N., Tang L., Grace J., Wong D., Con D., Sinclair M., Majumdar A., Kutaiba N. (2024). Statin, aspirin and metformin use and risk of hepatocellular carcinoma related outcomes following liver transplantation: A retrospective study. World J. Transplant..

[214] Campbell C., Wang T., McNaughton A.L., Barnes E., Matthews P.C. (2021). Risk factors for the development of hepatocellular carcinoma (HCC) in chronic hepatitis B virus (HBV) infection: A systematic review and meta-analysis. J. Viral Hepat..

[215] Zhou S.N., Zhang N., Liu H.H., Xia P., Zhang C., Song J.W., Fan X., Shi M., Jin L., Zhang J.Y. (2021). Skewed CD39/CD73/adenosine pathway contributes to B-cell hyperactivation and disease progression in patients with chronic hepatitis B. Gastroenterol. Rep..

[216] Sahra I., Regazzetti C., Robert G., Laurent K., Marchand-Brustel Y., Auberger P., Tanti J.-F., Giorgetti-Peraldi S., Bost F. (2011). Metformin, independent of AMPK, induces mTOR inhibition and cell-cycle arrest through REDD1. Cancer Res..

[217] Suhail M., Sohrab S.S., Kamal M.A., Azhar E.I. (2022). Role of hepatitis c virus in hepatocellular carcinoma and neurological disorders: An overview. Front. Oncol..

[218] Ferrín G., Guerrero M., Amado V., Rodríguez-Perálvarez M., De la Mata M. (2020). Activation of mTOR Signaling Pathway in Hepatocellular Carcinoma. Int. J. Mol. Sci..

[219] Kalender A., Selvaraj A., Kim S.Y., Gulati P., Brûlé S., Viollet B., Kemp B.E., Bardeesy N., Dennis P., Schlager J.J. (2010). Metformin, independent of AMPK, inhibits mTORC1 in a rag GTPase-dependent manner. Cell Metab..

[220] Wang Z., Jin W., Jin H., Wang X. (2014). mTOR in viral hepatitis and hepatocellular carcinoma: Function and treatment. BioMed Res. Int..

[221] Cichoż-Lach H., Michalak A. (2014). Oxidative stress as a crucial factor in liver diseases. World J. Gastroenterol..

[222] Maiers J.L., Chakraborty S. (2023). The Cellular, Molecular, and Pathologic Consequences of Stress on the Liver. Am. J. Pathol..

[223] Marycz K., Tomaszewski K.A., Kornicka K., Henry B.M., Wroński S., Tarasiuk J., Maredziak M. (2016). Metformin Decreases Reactive Oxygen Species, Enhances Osteogenic Properties of Adipose-Derived Multipotent Mesenchymal Stem Cells In Vitro, and Increases Bone Density In Vivo. Oxidative Med. Cell. Longev..

[224] Wang G., Wang Y., Yang Q., Xu C., Zheng Y., Wang L., Wu J., Zeng M., Luo M. (2022). Metformin prevents methylglyoxal-induced apoptosis by suppressing oxidative stress in vitro and in vivo. Cell Death Dis..

[225] Herman R., Kravos N.A., Jensterle M., Janež A., Dolžan V. (2022). Metformin and Insulin Resistance: A Review of the Underlying Mechanisms behind Changes in GLUT4-Mediated Glucose Transport. Int. J. Mol. Sci..

[226] Hammerstad S.S., Grock S.F., Lee H.J., Hasham A., Sundaram N., Tomer Y. (2015). Diabetes and Hepatitis C: A Two-Way Association. Front. Endocrinol..

[227] Lonardo A., Ballestri S., Guaraldi G., Nascimbeni F., Romagnoli D., Zona S., Targher G. (2016). Fatty liver is associated with an increased risk of diabetes and cardiovascular disease—Evidence from three different disease models: NAFLD, HCV and HIV. World J. Gastroenterol..

[228] Adinolfi L.E., Rinaldi L., Guerrera B., Restivo L., Marrone A., Giordano M., Zampino R. (2016). NAFLD and NASH in HCV Infection: Prevalence and Significance in Hepatic and Extrahepatic Manifestations. Int. J. Mol. Sci..

[229] Perazza F., Leoni L., Colosimo S., Musio A., Bocedi G., D’Avino M., Agnelli G., Nicastri A., Rossetti C., Sacilotto F. (2024). Metformin and the Liver: Unlocking the Full Therapeutic Potential. Metabolites.

[230] Hyun B., Shin S., Lee A., Lee S., Song Y., Ha N.J., Cho K.H., Kim K. (2013). Metformin Down-regulates TNF-α Secretion via Suppression of Scavenger Receptors in Macrophages. Immune Netw..

[231] Hegazy W.A.H., Rajab A.A.H., Abu Lila A.S., Abbas H.A. (2021). Anti-diabetics and antimicrobials: Harmony of mutual interplay. World J. Diabetes.

[232] Liu X., Yu P., Xu Y., Wang Y., Chen J., Tang F., Hu Z., Zhou J., Liu L., Qiu W. (2023). Metformin induces tolerogenicity of dendritic cells by promoting metabolic reprogramming. Cell. Mol. Life Sci. CMLS.

[233] Urbanowicz A., Zagożdżon R., Ciszek M. (2019). Modulation of the Immune System in Chronic Hepatitis C and During Antiviral Interferon-Free Therapy. Arch. Immunol. Ther. Exp..

[234] Li Z., Ding Q., Ling L.P., Wu Y., Meng D.X., Li X., Zhang C.Q. (2018). Metformin attenuates motility, contraction, and fibrogenic response of hepatic stellate cells in vivo and in vitro by activating AMP-activated protein kinase. World J. Gastroenterol..

[235] Yang J., Li S., Liu S., Zhang Y., Shen D., Wang P., Dang X. (2023). Metformin ameliorates liver fibrosis induced by congestive hepatopathy via the mTOR/HIF-1α signaling pathway. Ann. Hepatol..

[236] Kong L., Ma J., Dong L., Zhu C., Zhang J., Li J. (2024). Metformin exerts anti-liver fibrosis effect based on the regulation of gut microbiota homeostasis and multi-target synergy. Heliyon.

[237] Zeisel M.B., Fofana I., Fafi-Kremer S., Baumert T.F. (2011). Hepatitis C virus entry into hepatocytes: Molecular mechanisms and targets for antiviral therapies. J. Hepatol..

[238] Tsai W.L., Chang T.H., Sun W.C., Chan H.H., Wu C.C., Hsu P.I., Cheng J.S., Yu M.L. (2017). Metformin activates type I interferon signaling against HCV via activation of adenosine monophosphate-activated protein kinase. Oncotarget.

[239] Shojaeian A., Nakhaie M., Amjad Z., Boroujeni A., Shokri S., Mahmoudvand S. (2024). Leveraging metformin to combat hepatocellular carcinoma: Its therapeutic promise against hepatitis viral infections. J. Cancer Metastasis Treat..

[240] Zheng J., Woo S.L., Hu X., Botchlett R., Chen L., Huo Y., Wu C. (2015). Metformin and metabolic diseases: A focus on hepatic aspects. Front. Med..

[241] Yang T., Guan Q., Shi J.S., Xu Z.H., Geng Y. (2023). Metformin alleviates liver fibrosis in mice by enriching Lactobacillus sp. MF-1 in the gut microbiota. Biochim. Biophys. Acta Mol. Basis Dis..

[242] Pavlo P., Kamyshna I., Kamyshnyi A. (2023). Effects of metformin on the gut microbiota: A systematic review. Mol. Metab..

[243] Padilha M.D.M., Melo F.T.V., Laurentino R.V., da Silva A., Feitosa R.N.M. (2024). Dysregulation in the microbiota by HBV and HCV infection induces an altered cytokine profile in the pathobiome of infection. Braz. J. Infect. Dis..

[244] Schwenger K.J., Clermont-Dejean N., Allard J.P. (2019). The role of the gut microbiome in chronic liver disease: The clinical evidence revised. JHEP Rep. Innov. Hepatol..

[245] Liang H., Song H., Zhang X., Song G., Wang Y., Ding X., Duan X., Li L., Sun T., Kan Q. (2022). Metformin attenuated sepsis-related liver injury by modulating gut microbiota. Emerg. Microbes Infect..

[246] Di Vincenzo F., Del Gaudio A., Petito V., Lopetuso L.R., Scaldaferri F. (2024). Gut microbiota, intestinal permeability, and systemic inflammation: A narrative review. Intern. Emerg. Med..

[247] Zhang Y., Zhu X., Yu X., Novák P., Gui Q., Yin K. (2023). Enhancing intestinal barrier efficiency: A novel metabolic diseases therapy. Front. Nutr..

[248] Wang Y., Jia X., Cong B. (2024). Advances in the mechanism of metformin with wide-ranging effects on regulation of the intestinal microbiota. Front. Microbiol..

[249] Stein S.A., Lamos E.M., Davis S.N. (2013). A review of the efficacy and safety of oral antidiabetic drugs. Expert Opin. Drug Saf..

[250] Sacco M., Ribaldone D.G., Saracco G.M. (2023). Metformin and Hepatocellular Carcinoma Risk Reduction in Diabetic Patients with Chronic Hepatitis C: Fact or Fiction?. Viruses.

[251] Aziz K., Shahbaz A., Umair M., Sachmechi I. (2018). Treatment of Hepatitis C with Sofosbuvir, Velpatasvir and Voxilaprevir Decreases Hemoglobin A1c and Dependence on Anti-Glycemic Medications. Biomed. J. Sci. Tech. Res..

[252] Rosenthal E.S., Kottilil S., Polis M.A. (2016). Sofosbuvir and ledipasvir for HIV/HCV co-infected patients. Expert Opin. Pharmacother..

[253] Zhou J., Ke Y., Lei X., Wu T., Li Y., Bao T., Tang H., Zhang C., Wu X., Wang G. (2020). Meta-analysis: The efficacy of metformin and other anti-hyperglycemic agents in prolonging the survival of hepatocellular carcinoma patients with type 2 diabetes. Ann. Hepatol..

[254] Fujita K., Iwama H., Miyoshi H., Tani J., Oura K., Tadokoro T., Sakamoto T., Nomura T., Morishita A., Yoneyama H. (2016). Diabetes mellitus and metformin in hepatocellular carcinoma. World J. Gastroenterol..

[255] Arbuthnot P., Kew M. (2001). Hepatitis B virus and hepatocellular carcinoma. Int. J. Exp. Pathol..

[256] Shah N.J., Aloysius M.M., Sharma N.R., Pallav K. (2021). Advances in treatment and prevention of hepatitis B. World J. Gastrointest. Pharmacol. Ther..

[257] Xun Y.H., Zhang Y.J., Pan Q.C., Mao R.C., Qin Y.L., Liu H.Y., Zhang Y.M., Yu Y.S., Tang Z.H., Lu M.J. (2014). Metformin inhibits hepatitis B virus protein production and replication in human hepatoma cells. J. Viral Hepat..

[258] Yang Y.M., Kim S.Y., Seki E. (2019). Inflammation and Liver Cancer: Molecular Mechanisms and Therapeutic Targets. Semin. Liver Dis..

[259] Zhang Y., Wang H., Xiao H. (2021). Metformin Actions on the Liver: Protection Mechanisms Emerging in Hepatocytes and Immune Cells against NASH-Related HCC. Int. J. Mol. Sci..

[260] Ye J., Hu X., Wu T., Wu Y., Shao C., Li F., Lin Y., Feng S., Wang W., Zhong B. (2019). Insulin resistance exhibits varied metabolic abnormalities in nonalcoholic fatty liver disease, chronic hepatitis B and the combination of the two: A cross-sectional study. Diabetol. Metab. Syndr..

[261] Akter S. (2022). Non-alcoholic Fatty Liver Disease and Steatohepatitis: Risk Factors and Pathophysiology. Middle East J. Dig. Dis..

[262] Kawaguchi T., Taniguchi E., Itou M., Sakata M., Sumie S., Sata M. (2011). Insulin resistance and chronic liver disease. World J. Hepatol..

[263] García-Compeán D., Orsi E., Kumar R., Gundling F., Nishida T., Villarreal-Pérez J.Z., Del Cueto-Aguilera Á.N., González-González J.A., Pugliese G. (2022). Clinical implications of diabetes in chronic liver disease: Diagnosis, outcomes and management, current and future perspectives. World J. Gastroenterol..

[264] Pinyopornpanish K., Leerapun A., Pinyopornpanish K., Chattipakorn N. (2021). Effects of Metformin on Hepatic Steatosis in Adults with Nonalcoholic Fatty Liver Disease and Diabetes: Insights from the Cellular to Patient Levels. Gut Liver.

[265] de Oliveira S., Houseright R.A., Graves A.L., Golenberg N., Korte B.G., Miskolci V., Huttenlocher A. (2019). Metformin modulates innate immune-mediated inflammation and early progression of NAFLD-associated hepatocellular carcinoma in zebrafish. J. Hepatol..

[266] Malaekeh-Nikouei A., Shokri-Naei S., Karbasforoushan S., Bahari H., Baradaran Rahimi V., Heidari R., Askari V.R. (2023). Metformin beyond an anti-diabetic agent: A comprehensive and mechanistic review on its effects against natural and chemical toxins. Biomed. Pharmacother..

[267] Nevola R., Beccia D., Rosato V., Ruocco R., Mastrocinque D., Villani A., Perillo P., Imbriani S., Delle Femine A., Criscuolo L. (2023). HBV Infection and Host Interactions: The Role in Viral Persistence and Oncogenesis. Int. J. Mol. Sci..

[268] Zheng P., Dou Y., Wang Q. (2023). Immune response and treatment targets of chronic hepatitis B virus infection: Innate and adaptive immunity. Front. Cell. Infect. Microbiol..

[269] Xu L., Wang X., Chen Y., Soong L., Chen Y., Cai J., Liang Y., Sun J. (2021). Metformin Modulates T Cell Function and Alleviates Liver Injury Through Bioenergetic Regulation in Viral Hepatitis. Front. Immunol..

[270] Nguyen G., Park S.Y., Le C.T., Park W.S., Choi D.H., Cho E.H. (2018). Metformin ameliorates activation of hepatic stellate cells and hepatic fibrosis by succinate and GPR91 inhibition. Biochem. Biophys. Res. Commun..

[271] Su Y., Lu S., Hou C., Ren K., Wang M., Liu X., Zhao S., Liu X. (2022). Mitigation of liver fibrosis via hepatic stellate cells mitochondrial apoptosis induced by metformin. Int. Immunopharmacol..

[272] Su Y., Hou C., Wang M., Ren K., Zhou D., Liu X., Zhao S., Liu X. (2023). Metformin induces mitochondrial fission and reduces energy metabolism by targeting respiratory chain complex I in hepatic stellate cells to reverse liver fibrosis. Int. J. Biochem. Cell Biol..

[273] Li X., Wang X., Gao P. (2017). Diabetes Mellitus and Risk of Hepatocellular Carcinoma. BioMed Res. Int..

[274] Parikh P., Ryan J.D., Tsochatzis E.A. (2017). Fibrosis assessment in patients with chronic hepatitis B virus (HBV) infection. Ann. Transl. Med..

[275] Yang K., Song M. (2023). New Insights into the Pathogenesis of Metabolic-Associated Fatty Liver Disease (MAFLD): Gut-Liver-Heart Crosstalk. Nutrients.

[276] Ouyang J., Zaongo S.D., Zhang X., Qi M., Hu A., Wu H., Chen Y. (2021). Microbiota-Meditated Immunity Abnormalities Facilitate Hepatitis B Virus Co-Infection in People Living With HIV: A Review. Front. Immunol..

[277] Rosell-Díaz M., Petit-Gay A., Molas-Prat C., Gallardo-Nuell L., Ramió-Torrentà L., Garre-Olmo J., Pérez-Brocal V., Moya A., Jové M., Pamplona R. (2024). Metformin-induced changes in the gut microbiome and plasma metabolome are associated with cognition in men. Metab. Clin. Exp..

[278] Rosell-Díaz M., Fernández-Real J.M. (2024). Metformin, Cognitive Function, and Changes in the Gut Microbiome. Endocr. Rev..

[279] Ohtani N., Kawada N. (2019). Role of the Gut-Liver Axis in Liver Inflammation, Fibrosis, and Cancer: A Special Focus on the Gut Microbiota Relationship. Hepatol. Commun..

[280] Huang K.H., Lee C.H., Cheng Y.D., Gau S.Y., Tsai T.H., Chung N.J., Lee C.Y. (2022). Correlation between long-term use of metformin and incidence of NAFLD among patients with type 2 diabetes mellitus: A real-world cohort study. Front. Endocrinol..

[281] Ma S.J., Zheng Y.X., Zhou P.C., Xiao Y.N., Tan H.Z. (2016). Metformin use improves survival of diabetic liver cancer patients: Systematic review and meta-analysis. Oncotarget.

[282] Arvanitakis K., Koufakis T., Kalopitas G., Papadakos S.P., Kotsa K., Germanidis G. (2024). Management of type 2 diabetes in patients with compensated liver cirrhosis: Short of evidence, plenty of potential. Diabetes Metab. Syndr..

[283] Huang S.C., Kao J.-H. (2023). The interplay between chronic hepatitis B and diabetes mellitus: A narrative and concise review. Kaohsiung J. Med. Sci..

[284] Farfan Morales C., Cordero C., Osuna-Ramos J., Monroy Muñoz I., De Jesús-González L., Muñoz-Medina J., Hurtado Monzón A., Reyes-Ruiz J., Del Angel R. (2021). The antiviral effect of metformin on zika and dengue virus infection. Sci. Rep..

[285] Poglitsch M., Weichhart T., Hecking M., Werzowa J., Katholnig K., Antlanger M., Krmpotic A., Jonjic S., Hörl W.H., Zlabinger G.J. (2012). CMV late phase-induced mTOR activation is essential for efficient virus replication in polarized human macrophages. Am. J. Transplant..

[286] Rampersad S., Tennant P. (2018). Replication and Expression Strategies of Viruses. Viruses.

[287] Chen H., Zhou J., Chen H., Liang J., Xie C., Gu X., Wang R., Mao Z., Zhang Y., Li Q. (2022). Bmi-1-RING1B prevents GATA4-dependent senescence-associated pathological cardiac hypertrophy by promoting autophagic degradation of GATA4. Clin. Transl. Med..

[288] Combs J.A., Monk C.H., Harrison M.A.A., Norton E.B., Morris C.A., Sullivan D.E., Zwezdaryk K.J. (2021). Inhibiting cytomegalovirus replication through targeting the host electron transport chain. Antivir. Res..

[289] Nojima I., Eikawa S., Tomonobu N., Hada Y., Kajitani N., Teshigawara S., Miyamoto S., Tone A., Uchida H.A., Nakatsuka A. (2020). Dysfunction of CD8+ PD-1+ T cells in type 2 diabetes caused by the impairment of metabolism-immune axis. Sci. Rep..

[290] Poorghobadi S., Hosseini S.Y., Sadat S.M., Abdoli A., Irani S., Baesi K. (2024). The Combinatorial Effect of Ad-IL-24 and Ad-HSV-tk/GCV on Tumor Size, Autophagy, and UPR Mechanisms in Multiple Myeloma Mouse Model. Biochem. Genet..

[291] Berber E., Rouse B.T. (2022). Controlling Herpes Simplex Virus-Induced Immunoinflammatory Lesions Using Metabolic Therapy: A Comparison of 2-Deoxy-d-Glucose with Metformin. J. Virol..

[292] Wang X., Wang H., Yi P., Baker C., Casey G., Xie X., Luo H., Cai J., Fan X., Soong L. (2023). Metformin restrains ZIKV replication and alleviates virus-induced inflammatory responses in microglia. Int. Immunopharmacol..

[293] Singh S., Singh P.K., Suhail H., Arumugaswami V., Pellett P.E., Giri S., Kumar A. (2020). AMP-Activated Protein Kinase Restricts Zika Virus Replication in Endothelial Cells by Potentiating Innate Antiviral Responses and Inhibiting Glycolysis. J. Immunol..

[294] Velazquez-Cervantes M.A., López-Ortega O., Cruz-Holguín V.J., Herrera Moro-Huitron L., Flores-Pliego A., Lara-Hernandez I., Comas-García M., Villavicencio-Carrisoza O., Helguera-Reppeto A.C., Arévalo-Romero H. (2024). Metformin Inhibits Zika Virus Infection in Trophoblast Cell Line. Curr. Microbiol..

[295] Cheang N., Ting H., Koh H., Alonso S. (2021). In vitro and in vivo efficacy of Metformin against dengue. Antivir. Res..

[296] Bonglack E.N., Messinger J.E., Cable J.M., Ch’ng J., Parnell K.M., Reinoso-Vizcaíno N.M., Barry A.P., Russell V.S., Dave S.S., Christofk H.R. (2021). Monocarboxylate transporter antagonism reveals metabolic vulnerabilities of viral-driven lymphomas. Proc. Natl. Acad. Sci. USA.

[297] Hoppe-Seyler K., Herrmann A.L., Däschle A., Kuhn B.J., Strobel T.D., Lohrey C., Bulkescher J., Krijgsveld J., Hoppe-Seyler F. (2021). Effects of Metformin on the virus/host cell crosstalk in human papillomavirus-positive cancer cells. Int. J. Cancer.

[298] Hsu A.T., Hung Y.C., Fang S.H., D’Adamo C.R., Mavanur A.A., Svoboda S.M., Wolf J.H. (2021). Metformin use and the risk of anal intraepithelial neoplasia in type II diabetic patients. Color. Dis..

[299] Veeramachaneni R., Yu W., Newton J.M., Kemnade J.O., Skinner H.D., Sikora A.G., Sandulache V.C. (2021). Metformin generates profound alterations in systemic and tumor immunity with associated antitumor effects. J. Immunother. Cancer.

[300] Wilkie M.D., Anaam E.A., Lau A.S., Rubbi C.P., Vlatkovic N., Jones T.M., Boyd M.T. (2021). Metabolic Plasticity and Combinatorial Radiosensitisation Strategies in Human Papillomavirus-Positive Squamous Cell Carcinoma of the Head and Neck Cell Lines. Cancers.

[301] Sharma S., Munger K. (2020). KDM6A-Mediated Expression of the Long Noncoding RNA DINO Causes TP53 Tumor Suppressor Stabilization in Human Papillomavirus 16 E7-Expressing Cells. J. Virol..

[302] Curry J., Alnemri A., Philips R., Fiorella M., Sussman S., Stapp R., Solomides C., Harshyne L., South A., Luginbuhl A. (2023). CD8+ and FoxP3+ T-Cell Cellular Density and Spatial Distribution After Programmed Death-Ligand 1 Check Point Inhibition. Laryngoscope.

[303] Wong M. (2013). Mammalian target of rapamycin (mTOR) pathways in neurological diseases. Biomed. J..

[304] Bachman L.O., Zwezdaryk K.J. (2023). Targeting the Host Mitochondria as a Novel Human Cytomegalovirus Antiviral Strategy. Viruses.

[305] Li H., Ning X., Liu H., Chen Y., Ding X., Zhang H., Leng S. (2017). Metformin suppressed human cytomegalovirus (hCMV) replication and its potential molecular mechanisms in human fibroblasts. J. Immunol..

[306] Saavedra D., Añé-Kourí A.L., Barzilai N., Caruso C., Cho K.H., Fontana L., Franceschi C., Frasca D., Ledón N., Niedernhofer L.J. (2023). Aging and chronic inflammation: Highlights from a multidisciplinary workshop. Immun. Ageing I A.

[307] Samaniego L.A., Neiderhiser L., DeLuca N.A. (1998). Persistence and expression of the herpes simplex virus genome in the absence of immediate-early proteins. J. Virol..

[308] Movaqar A., Abdoli A., Aryan E., Jazaeri E.O., Meshkat Z. (2021). Metformin promotes autophagy activity and constrains HSV-1 replication in neuroblastoma cells. Gene Rep..

[309] Vink E.I., Smiley J.R., Mohr I. (2017). Subversion of Host Responses to Energy Insufficiency by Us3 Supports Herpes Simplex Virus 1 Replication during Stress. J. Virol..

[310] Nadhan R., Patra D., Krishnan N., Rajan A., Gopala S., Ravi D., Srinivas P. (2021). Perspectives on mechanistic implications of ROS inducers for targeting viral infections. Eur. J. Pharmacol..

[311] Buczyńska A., Sidorkiewicz I., Krętowski A.J., Adamska A. (2024). Examining the clinical relevance of metformin as an antioxidant intervention. Front. Pharmacol..

[312] Nguyen N.M., Chanh H.Q., Tam D.T.H., Vuong N.L., Chau N.T.X., Chau N.V.V., Phong N.T., Trieu H.T., Luong Thi Hue T., Cao Thi T. (2020). Metformin as adjunctive therapy for dengue in overweight and obese patients: A protocol for an open-label clinical trial (MeDO). Wellcome Open Res..

[313] Htun H.L., Yeo T.W., Tam C.C., Pang J., Leo Y.S., Lye D.C. (2018). Metformin Use and Severe Dengue in Diabetic Adults. Sci. Rep..

[314] Cao Y., Xie L., Shi F., Tang M., Li Y., Hu J., Zhao L., Zhao L., Yu X., Luo X. (2021). Targeting the signaling in Epstein-Barr virus-associated diseases: Mechanism, regulation, and clinical study. Signal Transduct. Target. Ther..

[315] Ruiz-Pablos M., Paiva B., Zabaleta A. (2023). Epstein-Barr virus-acquired immunodeficiency in myalgic encephalomyelitis-Is it present in long COVID?. J. Transl. Med..

[316] Chakravorty S., Afzali B., Kazemian M. (2022). EBV-associated diseases: Current therapeutics and emerging technologies. Front. Immunol..

[317] Yang T., You C., Meng S., Lai Z., Ai W., Zhang J. (2022). EBV Infection and Its Regulated Metabolic Reprogramming in Nasopharyngeal Tumorigenesis. Front. Cell. Infect. Microbiol..

[318] Adamson A.L., Le B.T., Siedenburg B.D. (2014). Inhibition of mTORC1 inhibits lytic replication of Epstein-Barr virus in a cell-type specific manner. Virol. J..

[319] Xia C., Liu C., He Z., Cai Y., Chen J. (2020). Metformin inhibits cervical cancer cell proliferation by modulating PI3K/Akt-induced major histocompatibility complex class I-related chain A gene expression. J. Exp. Clin. Cancer Res. CR.

[320] Hua Y., Zheng Y., Yao Y., Jia R., Ge S., Zhuang A. (2023). Metformin and cancer hallmarks: Shedding new lights on therapeutic repurposing. J. Transl. Med..

[321] Kim H.M., Kang M.J., Song S.O. (2022). Metformin and Cervical Cancer Risk in Patients with Newly Diagnosed Type 2 Diabetes: A Population-Based Study in Korea. Endocrinol. Metab..

[322] Kim K. (2024). Rethinking about Metformin: Promising Potentials. Korean J. Fam. Med..

[323] Kondo S., Yoshida K., Suzuki M., Saito I., Kanegae Y. (2014). Adenovirus-encoding virus-associated RNAs suppress HDGF gene expression to support efficient viral replication. PLoS ONE.

[324] Jiang H., Gomez-Manzano C., Rivera-Molina Y., Lang F.F., Conrad C.A., Fueyo J. (2015). Oncolytic adenovirus research evolution: From cell-cycle checkpoints to immune checkpoints. Curr. Opin. Virol..

[325] Prusinkiewicz M., Tu J., Dodge M., MacNeil K., Radko-Juettner S., Fonseca G., Pelka P., Mymryk J. (2020). Differential Effects of Human Adenovirus E1A Protein Isoforms on Aerobic Glycolysis in A549 Human Lung Epithelial Cells. Viruses.

[326] Lion T. (2014). Adenovirus infections in immunocompetent and immunocompromised patients. Clin. Microbiol. Rev..

[327] Cheng F., He M., Jung J.U., Lu C., Gao S.J. (2016). Suppression of Kaposi’s Sarcoma-Associated Herpesvirus Infection and Replication by 5′-AMP-Activated Protein Kinase. J. Virol..

[328] Yin H.C., Shao S.L., Jiang X.J., Xie P.Y., Sun W.S., Yu T.F. (2019). Interactions between Autophagy and DNA Viruses. Viruses.

